# Review targeted drug delivery systems for norcantharidin in cancer therapy

**DOI:** 10.1186/s12951-022-01703-3

**Published:** 2022-12-03

**Authors:** Bing-Tao Zhai, Jing Sun, Ya-Jun Shi, Xiao-Fei Zhang, Jun-Bo Zou, Jiang-Xue Cheng, Yu Fan, Dong-Yan Guo, Huan Tian

**Affiliations:** 1 State Key Laboratory of Research & Development of Characteristic Qin Medicine Resources (Cultivation), Shaanxi Key Laboratory of Chinese Medicine Fundamentals and New Drugs Research, Shaanxi Collaborative Innovation Center of Chinese Medicinal Resources Industrialization, Shaanxi University of Chinese Medicine, Xi’an, 712046 China; 2 Xi’an Hospital of Traditional Chinese Medicine, Xi’an, 710021 China

**Keywords:** Norcantharidin, Targeted drug delivery system, Passive targeting, Active targeting, Physicochemical targeting

## Abstract

Norcantharidin (NCTD) is a demethylated derivative of cantharidin (CTD), the main anticancer active ingredient isolated from traditional Chinese medicine Mylabris. NCTD has been approved by the State Food and Drug Administration for the treatment of various solid tumors, especially liver cancer. Although NCTD greatly reduces the toxicity of CTD, there is still a certain degree of urinary toxicity and organ toxicity, and the poor solubility, short half-life, fast metabolism, as well as high venous irritation and weak tumor targeting ability limit its widespread application in the clinic. To reduce its toxicity and improve its efficacy, design of targeted drug delivery systems based on biomaterials and nanomaterials is one of the most feasible strategies. Therefore, this review focused on the studies of targeted drug delivery systems combined with NCTD in recent years, including passive and active targeted drug delivery systems, and physicochemical targeted drug delivery systems for improving drug bioavailability and enhancing its efficacy, as well as increasing drug targeting ability and reducing its adverse effects.

**Graphical Abstract:**

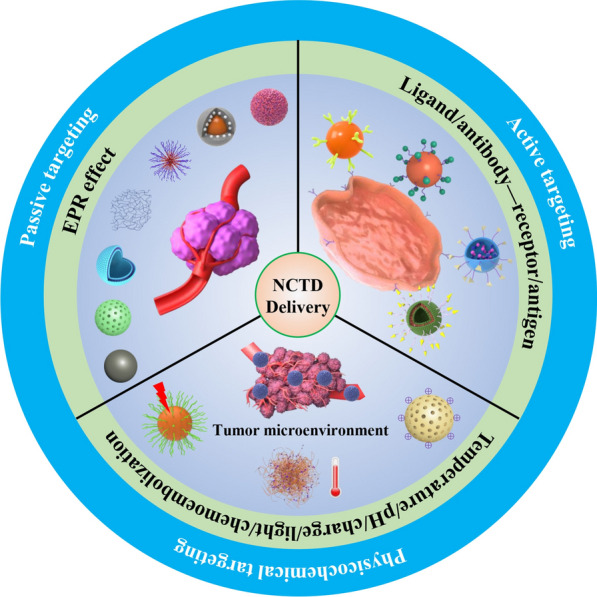

## Introduction

**Fig. 1:**
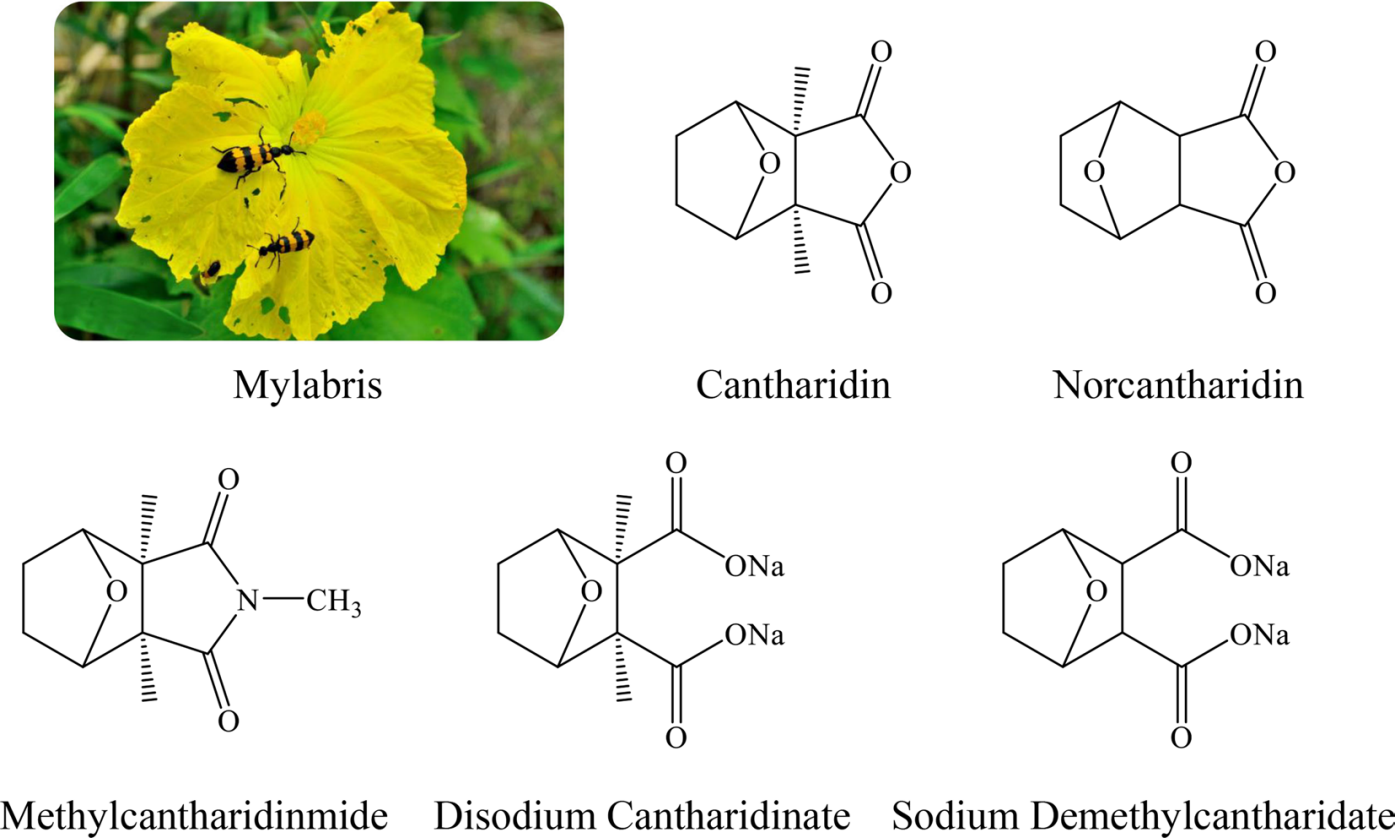
Structure of active ingredients in Mylabris and related antitumor preparations (Sodiun Demethylcantharidate for Injection, Sodium Demethylcantharidate Injection, Sodium Demethylcantharidate and Sodium Chloride Injection, Disodium Cantharidinate Injection, Disdium Cantharidinate and Vitamin B6 Injection, Cantharidatis Sodium Tablets, Mothylcantharidinmide Tablets, Demethylcantharidin Tablets)

Worldwide, cancer is a serious health problem and is the second leading cause of death [1]. Recently, traditional Chinese medicine has attracted more and more attention in cancer treatment. Mylabris is the dried body of the Chinese blister beetle, which is one of the earliest discovered medicines with antitumor effect in China. The species of Mylabris used in medicine usually are *Mylabris phalerata Pallas* and *Mylabris cichorii Linnaeus*. Cantharidin (CTD) is the main active ingredient of Mylabris [2]. CTD (exo, exo-2,3-dimethyl-7-oxobicyclo [2.2.1] heptane-2,3-dicarboxylic acid anhydride) is a colorless, odorless and shiny crystal. The molecular formula of CTD is C_10_H_12_O_4_, and the molecular weight is 196.2 g/mol. CTD has been confirmed to exert inhibitory effects on multiple types of cancers, such as liver cancer [3], acute myeloid leukemia [4], pancreatic cancer [5], gastric cancer [6], breast cancer [7], osteosarcoma [8] and lung cancer [9]. Moreover, it could recruit white blood cells, and may potentiate immune response [10, 11]. However, CTD is highly toxic, and oral and intravenous CTD have serious implications on both urinary system and digestive system [12–14]. In order to attenuate these adverse side effects, a series of CTD derivatives have been produced based on the structural optimization of CTD, such as norcantharidin (NCTD), disodium cantharidinate, sodium demethylcantharidate, and methylcantharidinmide (Fig. 1). These cantharidin derivatives retain the antitumor effect of CTD and reduce its toxic and side effects, showing good application advantages [15–18]. Currently, several antitumor chemicals based on the above CTD derivatives and several antitumor Chinese patent medicines containing Mylabris have been approved by the State Food and Drug Administration for the treatment of various solid tumors, especially liver cancer. Table 1 summarizes the names, dosage forms, compositions, indications, specifications, and usage of these marketed preparations in China.

**Table 1:** Antitumor marketed products containing Mylabris or its related active ingredients

Marketed product	Drug dosage form	Composition	Indications	Specification	Usage
Sodium Demethylcantharidate for Injection	Injection	Sodium Demethylcantharidate	For liver cancer, esophagus cancer, gastric and cardia cancer, lung cancer, and so on, and leukopenia. It can also be used as a preoperative drug for cancer or used in combined chemotherapy	10 mg/20 mg/30 mg (calculated as norcantharidin)	Intravenous injection; hepatic artery cannulation; intratumoral injection
Sodium Demethylcantharidate Injection	Injection	Sodium Demethylcantharidate	For liver cancer, esophagus cancer, gastric and cardia cancer, lung cancer, and so on, and leukopenia. It can also be used as a preoperative drug for cancer or used in combined chemotherapy	2 mL: 10 mg (calculated as norcantharidin)	Intravenous injection; hepatic artery cannulation; intratumoral injection
Sodium Demethylcantharidate and Sodium Chloride Injection	Injection	Sodium Demethylcantharidate, Sodium Chloride	For liver cancer, esophagus cancer, gastric and cardia cancer, lung cancer, and so on, and leukopenia. It can also be used as a preoperative drug for cancer or used in combined chemotherapy	250 mL: 30 mg (calculated as norcantharidin) and sodium chloride 2.25 g	Intravenous slow drip
Disodium Cantharidinate Injection	Injection	Disodium Cantharidinate	For primary liver cancer and other tumors and leukopenia. It can also be used for hepatitis, liver cirrhosis and hepatitis B virus carriers.	10 mL: 0.5 mg/2 mL: 0.1 mg/5 mL: 0.25 mg	Intravenous drip
Disdium Cantharidinate and Vitamin B6 Injection	Injection	Disodium Cantharidinate and Vitamin B6	For liver cancer, lung cancer and leukopenia. It can also be used for hepatitis, cirrhosis and hepatitis B virus carriers	5 mL: 0.05 mg/10 mL: 0.1 mg	Intravenous drip
Cantharidatis Sodium Tablets	Tablet	Disodium Cantharidinate	Antitumor drugs. For primary liver cancer and other tumors and leukopenia. It can also be used for hepatitis, liver cirrhosis and hepatitis B virus carriers.	0.5 mg	Oral
Methylcantharidinmide Tablets	Tablet	Methylcantharidinmide	For primary liver cancer	25 mg/10 mg	Oral
Compound Cantharidin Capsule	Capsule	Mylabris, Panax ginseng, Astragali Radix, Acanthopanax senticosus, Sparganii Rhizoma, Scutellaria barbata, Curcuma zedoaria, Corni Fructus, Ligustri Lucidi Fructus, bear bile powder, licorice	Removing blood and stasis, attacking poison and eroding sores. For primary liver cancer, lung cancer, rectal cancer, malignant lymphoma, gynecological malignant tumor, and so on.	0.25 g/capsule	Oral
Demethylcantharidin Tablets	Tablet	Norcantharidin	For liver cancer, esophageal cancer, gastric and cardia cancer, and so on, and leukopenia, hepatitis, cirrhosis, hepatitis B virus carriers.	5 mg	Oral
Aidi Injection	Injection	Mylabris, Panax ginseng, Astragali Radix, Acanthopanax senticosus	Clearing away heat and detoxifying, dissipating blood stasis and dissipating stagnation. For primary liver cancer, lung cancer, rectal cancer, malignant lymphoma, gynecological malignant tumor, and so on.	10 mL each	Intravenous drip
Delisheng Injection	Injection	Red ginseng, Astragali Radix, Bufonis Venenum, Mylabris	Nourish Qi and strengthen the body, relieve swelling and loose knot. For middle and advanced primary liver cancer with Qi deficiency and blood stasis syndrome, symptoms include mass in the right flank, persistent pain, abdominal distension, lack of appetite, and fatigue.	10 mL each	Intravenous drip
Ganning Tablets	Tablet	Mylabris, Arnebiae Radix, Glutinous rice	Clearing away heat and detoxifying, removing dampness, removing blood stasis and dissipating stagnation. It is used for the treatment of various acute and chronic hepatitis, especially for those with abnormal liver function and positive surface antigen in hepatitis B patients, and it can prevent hepatitis B from developing cancer.	Each tablet weighs 0.3 g	Oral
Hupo Zhitong plaster	Rubber plaster	Kaempferiae Rhizoma, Acorus Tatarinowii, Coptidis Rhizoma, Strychni Semen, Mylabris, Clematis chinensis Osbeck, Arisaematis Rhizoma, Bufonis Venenum, Amber oil, Basil oil, Peppermint oil, Star anise oil, Chinese cinnamon oil, Borneol, Camphor	Promote blood circulation and resolve phlegm, reduce swelling and dissipate knots, and relieve pain by dredging collaterals. For tumor pain, neuropathic pain, rheumatic arthralgia, bruises and blood stasis caused by phlegm and blood stasis	6 cm × 10 cm/piece	For external use, stick to the washed affected area

NCTD (7-oxabicyclo [2.2.1] heptane-2, 3-dicarboxylic anhydride), a chemically demethylated analog of CTD, was extracted from CTD [19], or was synthesized from furan and maleic anhydride [20]. The molecular formula of NCTD is C_8_H_8_O_4_, and the molecular weight is 168.150 g/mol. As an effective antitumor drug, NCTD has higher antitumor activity than CTD, and has been administered for years to treat cancer patients in China [21]. Whether NCTD is administered orally, or sodiun demethylcantharidate is administered by intravenous drip or intratumoral injection, the tumor growth can be effectively inhibited [22–25]. Moreover, NCTD/sodiun demethylcantharidate combined with radiotherapy, chemotherapy [oxaliplatin, fluorouracil, cisplatin, paclitaxel, gemcitabine, docetaxel, carboplatin, doxorubicin (Dox) and other chemotherapy drugs], iodine 125 seed implantation or transarterial chemoembolization can not only effectively improve the treatment effect of various cancers, such as esophageal cancer, colorectal cancer, gastric cancer, liver cancer, cervical cancer, non-small cell lung cancer, but also can effectively reduce the incidence of adverse reactions, such as leukopenia, neutropenia, thrombocytopenia, nausea and vomiting, bone marrow suppression, liver damage [26–39]; it can also improve the immune function of cancer patients by regulating T lymphocyte subsets and IgG levels, thereby improving the life quality of patients and prolonging the survival time of patients [40, 41]. Although NCTD greatly reduces the toxicity of CTD, there is still a certain degree of urinary toxicity, and organ toxicity in high-dose or long-term use [42, 43]. Moreover, the poor solubility, short half-life, fast metabolism, as well as high venous irritation and weak tumor targeting ability limit its wide clinical application [44–46].

Design of targeted drug delivery systems based on biomaterials and nanomaterials is one of the most feasible strategies to solve the aforementioned problems. Targeted drug delivery systems can effectively improve the solubility and in vivo drug distribution of poorly soluble drugs; nanoparticles are passively targeted to tumor cells through the enhanced penetration and retention (EPR) effect of solid tumors, or modified specific ligands or antibodies on the surface of nanoparticles are actively targeted to tumor cells, can also improve the selectivity of the drug to tumor cells, increase the concentration of the drug in the target area, reduce the distribution of the drug in the non-target site, and reduce adverse reactions [47–49]. Design endogenous tumor microenvironment-responsive drug delivery systems based on the special differences between tumor tissue and normal tissue microenvironment, and design exogenous stimulus-responsive drug delivery systems by utilizing the unique properties of the carrier itself, such as light, temperature, charge, and magnetism, can also effectively solve problem of in vivo localized drug release through responsive drug release by chemical bond cleavage or structural depolymerization of nanocarriers [50–53].

**Fig. 2:**
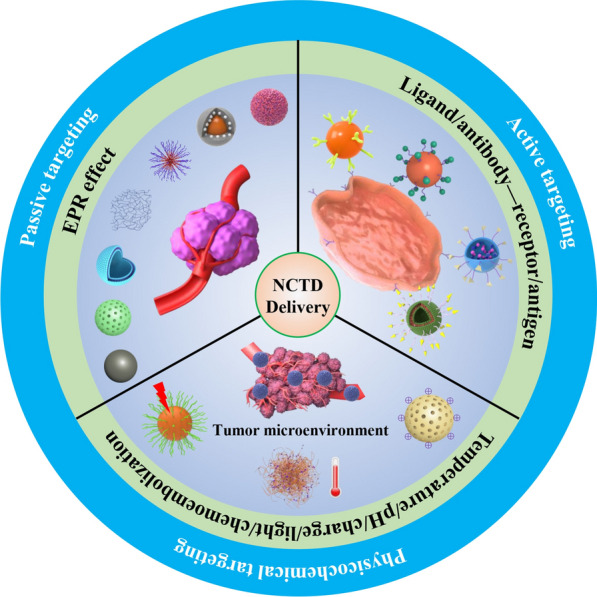
Schematic diagram of the classification of norcantharidin targeted drug delivery systems

In order to better exert the anticancer activity of NCTD, reduce toxicity, and change its pharmacokinetics and in vivo distribution characteristics, many researchers have adopted different targeted drug delivery systems, such as microspheres, microemulsions, liposomes, nanoparticles to overcome its clinical limitations. This review focused on the studies of targeted drug delivery systems combined with NCTD in recent years, including passive and active targeted drug delivery systems, and physicochemical targeted drug delivery systems for improving drug bioavailability and enhancing its efficacy, as well as increasing drug targeting ability and reducing its adverse effects, thereby providing new ideas for the clinical application of NCTD in the future (Fig. 2).

## Passive targeted drug delivery systems

Passive targeted drug delivery systems are drug delivery systems that passively enriche drugs in tumor tissue based on EPR effects at the tumor site, including liposomes, micelles, nanoparticles, microemulsion and self-microemulsion, chitosan (CS)-based drug delivery systems, microsphere, and so on. The EPR effect has been the rationale behind the field of nano-drug delivery systems for cancer treatment [54]. Passive targeted drug delivery systems of NCTD are summarized in Table 2.

**Table 2:** Passive targeted drug delivery systems of norcantharidin

Drug delivery system	Prescription (Method)	Characterization	Pharmacokinetics/tissue distribution/efficacy	Ref., year
NCTD liposomes	Drug/lipids ratio = 1: 20, phospholipid/cholesterol ratio = 2: 1, pH = 6.8 (Film hydration method)	Size: 360 nm, EE: 47.5%	-	[56], 2005
NCTD proliposome	1.0 g soybean lecithin, 0.823 g cholesterol, 85 mL double distilled water, 2.5 g trehalose (Ultrasound-nanomachine method and freeze-drying method)	Size: 580 nm, EE: (38.3 ± 0.06) %, zeta potential: -44.23 mV, viscosity: 1.83 mPa·s, freezing point depression value: 0.64 ℃	LD_50_: NCTD proliposome = 47.4 mg/kg, NCTD = 25.4 mg/kg; H22 tumor inhibition rate: NCTD (3 mg/kg, *ip.*, 1–7 day) = 37.6%, NCTD proliposome (10 mg/kg, *ip.*, 1, 4 day) = 43.8%, NCTD proliposome (10 mg/kg, *iv.*, 1, 4 day) = 48.4%	[57], 2006
NCTD proliposome	Drug/lipids = 0.346, cholesterol/ phospholipid = 0.038, 0.9% phospholipid, 0.8% NCTD (Ethanol injection method and freeze-drying method)	EE: 33.10%, pH: 7.8, repose angle: 30º	NCTD concentration in plasma, liver: NCTD proliposome > NCTD injection	[58], 2008
Disodium norcantharidate liposome	Drug/lipids ratio = 1: 20, phospholipid/cholesterol ratio = 8:1, water phase/oil phase = 1: 4 (Reverse evaporation method)	Size: 243.1 nm, zeta potential: -22.94 mV, pH: 7.54 ± 0.13, EE: (34.34 ± 1.21) %	Liposome/solution: t_1/2_, V1/F, CL/F, AUC, MRT ↑; targeting efficiency in kidney ↓; relative uptake efficiency in heart, liver, spleen, lung, kidney, brain, stomach, intestine, uterus > 1	[59], 2009
NCTD liposome	Phospholipid/drug quality ratio = 10: 1, phosphatide/cholesterol mass ratio = 5: 1 (Reverse film evaporation technique)	Size: (90.50 ± 2.40) nm, EE: (34.7 ± 1.3) %	-	[60], 2012
NCTD-loaded PEG-PLC diblock copolymer micelles	Drug/PEG-PLC diblock copolymer quality ratio = 0.0625: 1, solvent: tetrahydrofuran (Volatile dialysis method)	Size: (95.6 ± 10.1) nm, drug loading: (6.0 ± 0.3) %, EE: (79.1 ± 0.8) %	HepG2 cells (NCTD: IC_50_ = 26.00 µg/mL, micelles: IC_50_ = 22.13 µg/mL); A549 cells (NCTD: IC_50_ = 27.27 µg/mL, micelles: IC_50_ = 11.54 µg/mL); A2780 cells (NCTD: IC_50_ = 26.40 µg/mL, micelles: IC_50_ = 9.87 µg/mL); S180 tumor inhibition rate (*iv.*, 8 days): NCTD (2 mg/kg) = 47.5%, micelles (2, 4 mg/kg) = 61.36%, 77.63%	[62], 2012
NCTD-loaded poloxamer polymer micelles	200 mg poloxamer F127, 10 mg NCTD, solvent: absolute ethanol (Thin-film hydration method)	Size: 10.3 nm, EE: 98%, drug-loading coeffieient: 4.67%	-	[63], 2015
NCTD-loaded micelles	DSPE-PEG2000-MAL, NCTD, solvent: absolute ethanol	Size: (138.6 ± 45.8) nm, drug-loading rate: (2.82 ± 0.05) %, EE: (83.67 ± 1.78) %, zeta potential: -(12.75 ± 0.34) mV	A549 tumor inhibition rate (*iv.*, 1 time/2 days, 8 weeks): NCTD (1 mg/kg) = 54.78%, micelles (1 mg/kg) = 64.35%	[64], 2017
NCTD-loaded PLGA nanoparticle	25 mg PLGA, 2.5–25 mg NCTD (Interfacial deposition method)	Size: about 150 nm, EE: 95%	LD_50_: nanoparticle = 66.7 ± 3.9 mg/kg, NCTD = 25.4 ± 1.9 mg/kg; H22-H tumor inhibition rate: NCTD (3 mg/kg, *ip.*, 1–7 day) = 37.6%, nanoparticle (10 mg/kg, *ip.*, 1, 4 day) = 43.8%, nanoparticle (10 mg/kg, *iv.*, 1, 4 day) = 48.4%; LA795 tumor inhibition rate: NCTD (6 mg/kg, *ip.*, 1–7 day) = 36.3%, nanoparticle (16 mg/kg, *ip.*, 1, 4, 7 day) = 47.4%	[66], 2009
NCTD-loaded PLA-PEG nanoparticle	0.04 g PLA-PEG, 0.06 g NCTD (Phase separation method)	Size: (97.4 ± 14.5) nm, EE: (51.7 ± 1. 32) %	Inhibition rate of GBC-SD cells at IC_50_: NCTD = (23.14 ± 3.77) µg/mL, nanoparticle = (56.42 ± 9. 45) µg/mL	[68], 2007
NCTD-HPCS nanoparticle	NCTD: 2 mg/mL, HPCS: 1 mg/mL, tripolyphosphate: 1 mg/mL (Ionic crosslinking method)	Size: 95.15 ± 3.18 nm, EE: (23.68 ± 1.79) %, drug loading: (54.53 ± 2.61) %	BEL-7402 cells: NCTD: IC_50_ = (283.72 ± 4.55) µg/mL, nanoparticle: IC_50_ = (194.26 ± 3.69) µg/mL	[70], 2012
PVP-NCTD-NPs	0.1 g CS, 0.04 g NCTD, 7.5 mL of 1.2 mg/mL aqueous solution of TPP, 0.26 g PVP K_30_ (Ionic gelation between chitosan and sodium tripolyphosphate)	Size: (140.03 ± 6.23) nm; EE: (56.33 ± 1.41) %; drug-loading efficiency: (8.38 ± 0.56) %	PVP-NCTD-NP/NCTD (relative bioavailability): oral: 173.3%, *iv.*: 325.5%; targeted index (*iv.*): 1.168	[72], 2012
NCTD-SLN	Drug/stearic acid ratio = 0.3512, soybean lecithin: 125.10 mg/100 mL, poloxamer: 7.82 mg/mL, oil film/water phase volume ratio: 0.1286 (Thin film-ultrasonic dispersion method)	Size: (189 ± 6.0) nm, zeta potential: (-23.15 ± 0.17) mV, pH: 5.4-6.0, drug loading: 10.12%, EE: (55.4 ± 1.2) %	NCTD-SLN/NCTD solution in plasma: t_1/2_, AUC, MRT ↑, V_d_, CL ↓; NCTD-SLN/NCTD solution in liver: relative uptake rate = 1.59, targeting efficiency = 1.45, peak concentration ratio = 1.36	[74], 2007
NCTD-NLC	NCTD-NE (W/O): 10.5 mg NCTD, 1.6511 g ethyl oleate, 0.6018 g cremophor, 0.4521 g PEG 400, 0.3 mL water; NCTD-NLC: 1.0 mL NCTD-NE (W/O), 127.0 mg glyceryl monostearate, 102.1 mg lecithin, 51.0 mg glyceryl tripalmitate, 6.1 mg stearamide, 131.0 mg Tween-80, 20.0 mL dichloromethane, 5 mL water	-	NCTD concentration in liver and tumor (*iv.*): NCTD-NLC > NCTD; HepG2 cells: 100 µmol/L NCTD/NCTD-NLC (48 h), cell viability = 58.72%, 42.82%; cell apoptosis: 15.56%, 20.82%; HepG2 tumor inhibition rate (32.6 mg/mouse, *iv.*): NCTD = 19.15%, NCTD-NLC = 27.48%	[76], 2022
NCTD cubic liquid crystalline nanoparticle	Glyceryl monooleate/F127: 9: 1, 0.5 g NCTD, Glyceryl monooleate/water: 1: 500 (Emulsification method)	Size: 140 nm, zeta potential: -21 mV, EE: 45.33%	-	[78], 2017
NCTD-loaded mesoporous silica nanoparticle	NCTD, mesoporous silica nanoparticle (Midified Stober method and saturated solution adsorption method)	Size: about 140 nm (PDI < 0.3), zeta potential: about 35 mV, surface area: 1165.5 m^2^/g, cumulative pore volume: 2.16 cm^3^/g, pore size: 2.86 nm, drug loading rate: 12.88%	-	[80], 2018
Strontium/chitosan/hydroxyapatite/NCTD composite biomaterial	3 g CS, 42 g SrCl_2_, 12 g Ca(OH)_2_, 30 g KH_2_PO_4_, 5/6 g, 5/3 g, 5/2 g NCTD (Coprecipitation and freeze-drying method)	-	MG-63 cells (caspase-3 ↑, caspase-9 ↑, MMP-9 ↓); MC3T3-E1 cells (ALP ↓, runt-associated transcription factor 2 ↓, osteocalcin ↓, osteopontin ↑)	[84], 2020
DMCA-Zn1 NPs and DMCA-Zn2 NPs	DMCA-Zn1: NCTD (0.003 g, 0.0176 mmol), Zn(NO_3_)_2_·6H_2_O (0.004 g, 0.012 mmol); DMCA-Zn2: NCTD (0.006 g, 0.0356 mmol), Zn(NO_3_)_2_·6H_2_O (0.003 g, 0.010 mmol); DMCA-Zn1 NPs: 2 mg DMCA-Zn1 crystals, 8 mg F127, 0.1 mL deionized water (A process of mechanical grinding, ultrasonic treatment and filtration)	Size: DMCA-Zn1 NPs: around 190 nm, DMCA-Zn2 NPs: around 162 nm	Fluorescence intensity of Hep3B xenograft mice (*iv.*): liver and tumor: Nile red < DMCA-Zn1 NPs < DMCA-Zn2 NPs; kidneys: Nile red > DMCA-Zn2 NPs > DMCA-Zn1 NPs; IC_50_ of HepG2 and Hep3B cells: DMCA-Zn1 NPs and DMCA-Zn2 NPs < NCTD; cytotoxicity to L929 normal cells: DMCA-Zn1 NPs and DMCA-Zn2 NPs < NCTD Hep3B tumor inhibition rate: NCTD = (54.89 ± 5.84) %, DMCA-Zn1 NPs = (67.72 ± 2.18) %, DMCA-Zn2 NPs = (62.96 ± 6.94) %; nanoparticle: no obvious liver and kidney injury (H&E and ALP, ALT, AST, BUN, UA)	[87], 2022
NCTD nanosuspension	22% hydroxypropyl cellulose-SL, 1% sodium dodecyl sulfate, 5 g NCTD (Wet media milling method)	Size: (325.4 ± 4.1) nm, PDI: 0.184 ± 0.009, zeta potential: (-32.5 ± 1.8) mV	-	[88], 2019
NCTD-loaded W/O microemulsion	7% water, 45% soybean lecithin/ethanol (2: 1), 48% ethyl oleate	Size: (44.5 ± 8.6) nm	NCTD microemulsion/injection in plasma: AUC, MRT, t_1/2_ ↑, V_d_, CL ↓; NCTD microemulsion/injection in liver: overall targeting efficiency = 6.10 ± 0.15, 3.66 ± 0.14, targeting index = 3.55, relative overall targeting efficiency = 0.67; kidney: overall targeting efficiency = 0.03 ± 0.01, 0.06 ± 0.05	[90], 2005
NCTD-SNEDDS	50% Ethyl Oleate, 35% Cremophor EL, 15% ethylene glycol, 10 mg NCTD	Size: 36.31 nm, PDI: 0.05	-	[92], 2017
NCTD solid self-microemulsion	NCTD, polyoxyethylene hydrogenated castor oil, 1,2-propylene glycol, castor oil, 0.08% sodium dodecyl sulfate aqueous solution, ethyl cellulose, SiO_2_ (Spherulite technology one-step curing method)	Size: 22.76 nm, zeta potential: -2.77 mV, EE: 77.39%, yield: 84.5%	-	[93], 2017
NCTD-conjugated chitosan conjugates (NCTD-CSs)	1 g chitosan (6.2 mmol calculated as glucosamine units), 15 mL MeSO_3_H, NCTD (1.0, 3.0 equiv/glucosamine units of chitosan) (Covalent attachment of NCTD to chitosan using the MeSO_3_H as reaction solvent)	NCTD-CS1/ NCTD-CS2: DS = 60.2% and 97.9%, NCTD content = 38.4%, 48.4%, water solubility = 50.8, 74.2 mg/mL	MGC80-3 cells: NCTD/NCTD-CS1/ NCTD-CS2: IC_50_ = 5.43, 28.2, 48.5 µg/mL; early and late apoptotic cells (48 h): NCTD = (8.03 ± 0.16) %, (21.9 ± 1.01) %, NCTD/CS1 = (12.8 ± 0.18) %, (40.2 ± 5.3) %	[94], 2013
NCTD-CS	5.04 g NCTD, 1.61 g CS (Alcoholysis reaction)	The degrees of NCTD grafting of NCTD-CS (mol/monomer mol): 89.6%, NCTD mass fractions: 48.0%	IC_50_ of NCTD, NCTD-CS on ECA-109 cell (48 h): (9.4 ± 0.9), (168.8 ± 8.9) mg/mL, on EMT6 cell: (3.1 ± 0.3), (90.7 ± 8.1) mg/mL; induce cell apoptosis and arrest cell cycle at the S phase; activate caspase-8 and caspase-3; EMT6 tumor inhibition rate (*iv.*): NCTD (10 mg/kg, once daily, 8 days) = (35.87 ± 6.25) %, NCTD-CS [1 and 5 day, 83 mg/kg (40 mg/kg NCTD-equivalent dose)] = (45.82 ± 12.12) %	[95], 2014
NCTD-conjugated hydroxypropyltrimethyl ammonium chloride chitosan derivatives (NCTD-HACCs)	1 g HACC, 15 mL MeSO_3_H, NCTD (1.0, 2.0 equiv/glucosamine units of HACC)	NCTD-HACC1/ NCTD-HACC2: DS = 12.2%, 24.8%, NCTD content = 9.29%, 17.0%, water solubility = 18.8, 26.2 mg/mL	In vivo NIR fluorescence real-time imaging (*iv.*): tumor > heart, liver, spleen and lung (except kidney); S180 tumor inhibition rate (one injection each day, 7 days): NCTD = 25.41%, HACC = 47.57%, NCTD-HACC2 = 42.70%	[97], 2013
NCTD-conjugated carboxymethyl chitosan conjugate (CNC)	1 g CMCS, NCTD (1.0 equiv., 0.7 g) (Chemical grafting technique)	DS = 30.10%, NCTD content = 20.05%	CNC/NCTD: AUC, t_1/2_, MRT ↑, CL, V_d_ ↓; relative uptake efficiency: liver, spleen = 1.438, 1.585 (> 1); heart, kidney = 0.790, 0.714 (< 1); BEL-7402 cells: CNC: enhance cytotoxicity compared with free NCTD, inhibit migration, induce apoptosis; H22 tumor inhibition rate (*ip.*, every other day for 12 days): NCTD (6.524 mg/kg) = 30.27%, CNC (32.62, 16.31 and 8.16 mg/kg) = 49.65%, 56.20%, 47.73%; TNF-α, IFN-γ ↑; heart, spleen, kidney toxicity ↓	[98], 2019
NCTD-conjugated carboxymethyl chitosan (CMCS-NCTD)	CMCS, NCTD (Chemical grafting technique)	DS = 30.81%, NCTD content = 20.05%	H22 tumor: CMCS-NCTD (3.12, 6.25, 12.5 mg/kg, *ip.*, every other day for 12 days): liver index ↓; TNF-α, IFN-γ, TIMP-1, E-cadherin ↑; ALT, AST, VEGF, MMP-9 ↓; SOD, GSH-Px ↑	[99], 2017
CMCS-NCTD	1.0 equiv./glucosamine units of CMCS, NCTD (Chemical grafting technique)	DS = 30.10%, NCTD content = 20.05%	A549 cells: CMCS-NCTD: obvious cytotoxicity, inhibit migration; Lewis lung carcinoma metastasis model, tumor inhibition rate (*ip.*, every other day for 14 days): NCTD (6.524 mg/kg) = 35.39%, CMCS-NCTD (32.62, 16.31 and 8.16 mg/kg) = 64.58%, 50.57%, 47.71%; median survival time: NCTD = 30 days, CMCS-NCTD (32.62 mg/kg) = 39 days; VEGF, MMP-9 ↓, TIMP-1 ↑	[100], 2019
CNC	0.2 g CMCS, NCTD (1.0 equiv.) (Amidation reaction)	NCTD content = 20.05%	SGC-7901 cells: CNC: inhibit proliferation, anti-angiogenesis effect, induce apoptosis; SGC-7901 tumor inhibition rate (*iv.*, every other day for 24 days): NCTD (6.524 mg/kg) = 40.46%, CMCS-NCTD (32.62, 16.31 mg/kg) = 59.57%, 50.64%; the density of positive microvessels in NCTD, high and low dose of CNC group were decreased by 13.27%, 26.39% and 20.51%, compared with that of control group; TNF-α, Bax, Caspase-3 ↑, CD34, VEGF, MMP-2, MMP-9, Bcl-2 ↓	[101], 2019
NCTD-PVA	5.04 g NCTD, 1.76 g PVA (Alcoholysis reaction)	The degrees of NCTD grafting of NCTD-CS (mol/monomer mol): 10.4%, NCTD mass fractions: 38.8%	IC_50_ of NCTD, NCTD-CS on ECA-109 cell (48 h): (9.4 ± 0.9), (55.3 ± 3.0) mg/mL, on EMT6 cell: (3.1 ± 0.3), (30.5 ± 5.4) mg/mL; induce cell apoptosis and arrest cell cycle at the S phase; activate caspase-8 and caspase-3; EMT6 tumor inhibition rate (*iv.*): NCTD (10 mg/kg, once daily, 8 days) = (35.87 ± 6.25) %, NCTD-PVA [1, 3 5 and 7 day, 52 mg/kg (20 mg/kg NCTD-equivalent dose)] = (56.17 ± 11.34) %	[95], 2014
NCTD-loaded lipid microsphere	A mixture of soybean oil and MCT 10 g; lecithin 1.2 g; Tween 80 20 mg, glycerol 2.5 g, DL-α-tocopherol 300 mg, sodium oleate 30 mg; EDTA 20 mg, NCTD 200 mg, doubly distilled water, qs 100.0 g (High-pressure homogenization process)	2 mg/mL NCTD, zeta potential: about − 38 mV	-	[45], 2006
NCTD-loaded lipid microsphere	Medium-chain triglyceride oil (MCT) (7.5%, w/v), Long-chain triglyceride (LCT) (2.5%, w/v), Egg phospholipids PL-100 M (3.6%, w/v), NCTD (0.2%, w/v); Poloxamer 188 (Pluronic F68) (0.4%, w/v), glycerin (2.5%, w/v), sodium oleate (0.03%, w/v) (Homogenization method)	Size: 167.4 ± 63.1 nm, zeta potential: -31.6 mV, EE: 84.9%	Microsphere/injection: no significant differences in pharmacokinetic parameters; the content and AUC of NCTD in heart: microsphere < injection; A549, BEL7402, BCAP-37 tumor inhibition rate (*iv.*, once a week): A549: injection (2.5 mg/kg) = 68.7%, microsphere (1.25, 2.5, 5.0 mg/kg) = 39.2%, 65.6%, 73.1%; BEL7402: injection (2.5 mg/kg) = 65.9%, microsphere (1.25, 2.5, 5.0 mg/kg) = 58.9%, 64.2%, 70.4%; BCAP-37: injection (2.5 mg/kg) = 65.9%, microsphere (1.25, 2.5, 5.0 mg/kg) = 56.3%, 57.3%, 70.7%; LD_50_ and 95% confidence limit for female mice and male mice: injection = 10.10 (8.33–13.10), 8.93 (6.92–11.77) mg/kg; microsphere = 15.67 (13.61–17.58), 16.64 (15.14–18.25) mg/kg; white blood cell count (WBC): microsphere = (18.5 ± 3.4 × 10^9^ L^− 1^), injection = (15.6 ± 2.4 × 10^9^ L^− 1^), NCTD = (11.8 ± 2.4 × 10^9^ L^− 1^); cardiac and renal toxicity: injection = 66.7% (20/30), 73.3% (22/30), microsphere = no obvious damage to the heart, 33.3% (10/30); no hemolysis or erythrocyte agglutination; no obvious intravenous irritation; no hypersensitivity reactions	[103], 2012
NCTD-loaded lipid microsphere (NPCLM)	NCTD-phospholipid complex (NPC): phospholipids (E80), cholesterol, NCTD; MCT 10% (w/v), oleic acid 0.06% (w/v), 2.5% (w/v) glycerol, 0.4% (w/v) F-68, 0.04% (w/v) EDTA, 0.8% (w/v) PL-100 M (Concentrated homogenization method and phospholipid complex method)	Size: (173.2 ± 41.6) nm, zeta potential: -34.54 mV, EE: (84.6 ± 0.62) %, pH: 7.69, content: (99.53 ± 0.11) %	Relative tissue exposure in liver, spleen, lung and kidney: NPCLM/injection = 1.67, 1.49, 1.06 and 0.96	[104], 2014
NCTD encapsulated albumin microspheres	about 30 mg NCTD, 2.5 mL diethyl ether, castor oil (25 mL) containing a surfactant (0.25 g span-80), 25% glutaraldehyde solution, 1 mL aqueous mannitol (20%, w/v) (Emulsion cross-linking method)	Size: (13.3 ± 0.4) µm, EE: (54.3 ± 4.18) %, PDI: 0.129 ± 0.039, zeta potential: -(12.1 ± 0.8) mV	Pharmacokinetic parameters: microsphere/injection: AUC, t_1/2_, MRT ↑, CL ↓; target index: liver, spleen = 3.49, 1.03 (> 1); heart, kidney = 0.79, 0.92 (< 1); no histological change occurred to the rat liver	[106], 2015
NCTD-loaded chitosan microsphere	Liquid paraffin, Span-80, formaldehyde, chitosan, NCTD (Emulsification cross-linking process)	Size: (25 ± 10) µm, drug-loading rate: (15.08 ± 2.85) %, EE: (57.80 ± 1.35) %	-	[107], 2008
NCTD loaded-emulsion-hybrid nanoparticle (NLEH)	NCTD phospholipid complexes [phospholipids (E80), cholesterol and NCTD (10:2.5:1)], MCT, oleic acid, glycerol, Poloxamer 188 and PL-100 M (High-pressure homogenization method)	Size: (163.8 ± 1.082) nm, PDI: 0.084, zeta potential: -(38.0 ± 7.11) mV, EE: 89.3%, drug loading: 2 mg/mL, pH: about 7.40	Cellular uptake ↑; inhibit H22 cell proliferation, induce cell apoptosis ↑; NLEH/NCTD solution in plasma: AUC, t_1/2_ ↑, CL ↓; NLEH/NCTD solution: targeting efficiency in tumor, liver and spleen = 1.19, 1.40, 1.21 > 1, targeting efficiency in kidneys, heart = 0.77, 0.73 < 1; enhance tumor penetration; H22 tumor inhibition rate (*iv.*, twice a week, 3 weeks, 2.7 mg/kg): NLEH > NCTD solution; improve immunity: the leukogenic effect, the spleen index ↑	[108], 2022

### Liposome-based NCTD delivery

Liposomes are spherical vesicles created by a lipid bilayer of phospholipids. Due to their weak immunogenic response and good biocompatibility, liposomes have emerged as a promising nano-drug delivery system [55]. Wu et al. [56] prepared a NCTD liposome by using film hydration method. The ratio of drug to lipids was 1: 20, the ratio of phospholipid to cholesterol was 2: 1, and pH of water phase was 6.8. The average particle size of the prepared liposomes is 360 nm, and the encapsulation efficiency (EE) reached 47.5%. Miao et al. [57] prepared a powdered NCTD proliposome with average size of 580 nm, EE of (38.3 ± 0.06) % and zeta potential of -44.23 mV. It exhibited good antitumor effect by intraperitoneal injection and tail vein injection on H22 tumor-bearing mice, with tumor inhibition rate of 43.8% and 48.4%, respectively. Moreover, the LD_50_ of NCTD proliposome is 47.4 mg/kg, and its toxicity is much lower than that of free NCTD (LD_50_ = 25.4 mg/kg). Liu et al. [58] also prepared a NCTD liposome by using ethanol injection method, and then freeze-dried to prepare the NCTD proliposome. The optimal preparation process was as follows: the ratio of drug to lipids was 0.346, the ratio of cholesterol to phospholipid was 0.038, and the content of phospholipid was 0.9%. The EE of proliposome after dispersion was 33.10%. Comparing the pharmacokinetic behavior of NCTD injection and NCTD proliposomes, it is proved that the distribution of the drug in liver is significantly increased. Therefore, the preparation of NCTD liposomes can make the drug more concentrated in the liver to exert its effects, while reducing adverse reactions. Moreover, Zhang et al. [59] prepared a disodium norcantharidate liposome by using reverse evaporation method, with average size of 243.1 nm, zeta potential of -22.94 mV, pH value of 7.54 ± 0.13 and EE of (34.34 ± 1.21) %. The disodium norcantharidate solution was completely released within 1 h, while the disodium norcantharidate liposome could continue to release for 12 h. In addition, the liposome improved the circulation time of the drug in the blood, increased the accumulation of the drug in the tumor tissue, and improved the targeting of the drug in various tissues, especially the uterus, and also reduced the toxicity to the kidneys. Besides, Gu et al. [60] prepared a NCTD liposomes by using reverse film evaporation technique. The phospholipid-drug quality ratio was 10:1, the phosphatide-cholesterol mass ratio was 5:1, and ultrasonic frequency was 10 times. The prepared liposome had a particle size of (90.50 ± 2.40) nm and an EE of (34.7 ± 1.3) %, and have significant in vitro slow-release characteristics.

### Micelle-based NCTD delivery

Polymeric micelles are self-assembled nanoparticles with a hydrophilic shell core and a hydrophobic core formed by self-assembly of amphiphilic polymers. The hydrophilic shell of micelles can provide steric stability and avoid the rapid uptake of the reticuloendothelial system, thereby prolonging the circulation time of drugs in the body; while the hydrophobic core has good compatibility with the encapsulated hydrophobic drugs, which endows the micelles with high drug-carrying capacity and can control the release of the drug [61]. Based on the above advantages, polymeric micelles have become an excellent antitumor drug delivery system.

Chen et al. [62] prepared a NCTD polymer micelle by volatile dialysis method, with a particle size of (95.6 ± 10.1) nm, a drug loading of (6.0 ± 0.3) %, and an EE of (79.1 ± 0.8) %. After 72 h, the amount of drug released from the micelles at pH 6.5, 7.0 and 7.4 was (83.4 ± 2.5) %, (80.0 ± 1.6) % and (72.0 ± 1.5) %, respectively, indicating that the drug-loaded micelles could release more drugs in the tumor slightly acidic environment. Compared with NCTD injection (47.50%), the same dose (2 mg/kg) of drug-loaded micelles had a tumor inhibition rate of 61.36% in S180 tumor model, and the tumor inhibition rate of 4 mg/kg drug-loaded micelles reached 77.63%. Yin et al. [63] prepared a NCTD-loaded polymeric micelles by thin-film hydration method with poloxamer F127 as the drug carrier. This polymeric micelle has an average particle size of 10.3 nm, EE of 98% and drug-loading coeffieient of 4.67%. Furthermore, Wang et al. [64] prepared a NCTD nano-micelle using the triblock copolymer distearyl phosphatidylethanolamine-polyethylene glycol-maleimide as the carrier. This nano-micelle has a particle size of (138.6 ± 45.8) nm, EE of (83.67 ± 1.78) %. Compared with NCTD injection (54.78%), the same dose (1 mg/kg) of drug-loaded micelles enhanced the antitumor effect in A549 tumor model, with a tumor inhibition rate of 64.35%.

### Nanoparticle-based NCTD delivery

In recent years, biodegradable polymers have been playing an important role in its functions of targeted drug delivery and controlled drug release. Poly (lactic-co-glycolic acid) (PLGA) is a polymer of poly (lactic acid) (PLA) and poly (glycolic acid) (PGA) that has been approved by the FDA for the treatment of human diseases. The polymer is non-toxic, non-irritating, and has good biocompatibility and biodegradability. PLGA nanoparticles can increase anticancer drugs solubility and stability in the biological milieu, and can control the slow and sustained release of the drug according to the design, thereby prolonging the circulation time of the drug in vivo and improving the bioavailability [65]. Zeng et al. [66] prepared a NCTD-loaded PLGA nanoparticle. The NCTD release from the nanoparticle showed biphasic profile with an initial rapid and a following slower release phase for more than 10 days. Compared with NCTD, the NCTD-PLGA nanoparticles showed better antitumor efficacy in mice models bearing ascites hepatoma and pulmonary adenocarcinoma. Moreover, NCTD-PLGA nanoparticles had no obvious side effects at LD_50_ dose level [(66.7 ± 3.9) mg/kg], while NCTD induced severe prostration, apathy, and catatonia at LD_50_ dose level [(25.4 ± 1.9) mg/kg]. PLA-polyethylene glycol (PLA-PEG) amphiphilic block copolymer as a drug carrier could also increase the drug loading of hydrophobic drugs, reduce the burst effect, extend blood circulation time and improve the bioavailability of drugs [67]. Ren et al. [68] prepared a NCTD nanoparticle using PLA-PEG as carrier by phase separation method. Compared with the naked drug, NCTD nanoparticle has good sustained-release property and can more effectively inhibit the growth of GBC-SD cell for 48 h. CS, obtained through the deacetylation of chitin, is an avirulent, biodegradable natural cationic polymer with good biocompatibility. CS and its derivatives have been widely used in the pharmaceutical industry as carriers for drug delivery, which could control drug release, improve drug efficacy, reduce drug side effects, increase drug stability and improve the targeting capabilities [69]. Feng et al. [70] prepared a NCTD hydroxypropyl-CS nanoparticle by ionic crosslinking method. Compared with the original drug, the drug-loaded nanoparticles have obvious sustained-release properties in vitro, and the release time can be extended to about 3 times of the original drug. The IC_50_ of free NCTD on liver cancer cells BEL-7402 was (283.72 ± 4.55) µg/mL, while the IC_50_ of the prepared nanoparticle was (194.26 ± 3.69) µg/mL, which was about 30% lower than that of free NCTD. As an amphiphilic polymer, polyvinylpyrrolidone-K_30_ (PVP-K_30_) could be used as a coating material for nanoparticles to improve the nanoparticles stability [71]. In order to enhance the stability of NCTD CS nanoparticles, Ding et al. [72] also prepared a PVP-K_30_-coated NCTD CS nanoparticles (PVP-NCTD-NPs), which showed a relative bioavailabilities of 173.3% and 325.5% by *p.o.* and *i.v.* administration, respectively, than in the NCTD group. The half-life (t_1/2_) increased and the clearance (CL) obviously decreased. Besides, the PVP-NCTD-NP improved the liver targeting effect of NCTD.

Solid Lipid Nanoparticle (SLN) is a solid colloidal drug delivery system with a particle size of 10-1000 nm formed by solid natural or synthetic lipid-encapsulated drugs. It is the first generation of lipid nanoparticles [73]. It can effectively improve the solubility and bioavailability of NCTD. Tian et al. [74] prepared a NCTD-loaded SLN (NCTD-SLN) by thin film-ultrasonic dispersion method. The NCTD-SLN significantly improved the bioavailability of free NCTD in vivo. Moreover, the relative uptake rate of NCTD-SLN to NCTD solution in liver tissue was 1.59, the targeting efficiency was 1.45, and the peak concentration ratio was 1.36, indicating that NCTD-SLN improved the targeting of free drug in liver tissue. Nanostructured lipid carrier (NLC) is a class of nanoparticle prepared by mixing solid and liquid lipids on the basis of SLN, which improves the disadvantages of easy leakage and low drug-loading capacity of SLN, and can prolong the circulation time and improve the therapeutic effect by increasing the stability of the drug and producing a sustained-release effect [75]. Yan et al. [76] prepared a NCTD-loaded NLC (NCTD-NLC). Compared with free NCTD, NCTD-NLC promoted apoptosis of HepG2 cells, and enhanced the antitumor effect via tail vein injection in HepG2 tumor model, with a tumor inhibition rate of 27.48%. Cubic liquid crystalline nanoparticle is a thermodynamically stable dispersion system spontaneously formed by amphiphilic lipid materials and suitable surfactants in water. The special structure and properties of cubic liquid crystal enable it to simultaneously encapsulate water-soluble, lipid-soluble and amphiphilic drug molecules, thereby effectively improving drug solubility, release rate and bioavailability [77]. Li et al. [78] prepared a NCTD cubic liquid crystalline nanoparticle by emulsification method. The NCTD solution was basically released completely within 4 h, while the NCTD cubic liquid crystalline nanoparticle released about 80% of the drug within 12 h, which had an obvious sustained-release effect.

As an inorganic nanomaterial, mesoporous silica nanoparticle (MSN) has unique and excellent properties such as large specific surface area, adjustable pore size, ordered pore structure, good stability, and high drug loading capacity, which can achieve controlled drug release, and then improve the therapeutic effect and reduce adverse reactions [79]. Xiong et al. [80] prepared a NCTD-loaded MSN by modified Stober method and saturated solution adsorption method. Free NCTD was released rapidly, and the release amount within 4 h was 90.6%, while the release rate of drug loaded MSN was 83.34% in 12 h with the sustained-release properties.

Hydroxyapatite can deliver antitumor drugs, and its composite material hydroxyapatite/CS can repair bone defects [81, 82]. Strontium (Sr), a trace element in the human body, has been found to have the ability to promote bone formation and inhibit osteoclasts, which have positive effects on improving the osteogenic activity of the composite [83]. Huang et al. [84] developed a novel Sr/CS/hydroxyapatite/NCTD composite biomaterial by coprecipitation and freeze-drying method. The composite biomaterial had good biocompatibility, which do well in antitumor properties by upregulating the expression of caspase-3/-9, and downregulating the expression of matrix metallopeptidase (MMP)-9. In addition, the composite material effectively promoted the mineralization of osteoblasts by downregulating the expression of alkaline phosphatase (ALP), runt-associated transcription factor 2, and osteocalcin, and upregulating the expression of osteopontin. In summary, the composite showed good potential for treating osteosarcoma and repairing tumor-related bone defects.

**Fig. 3:**
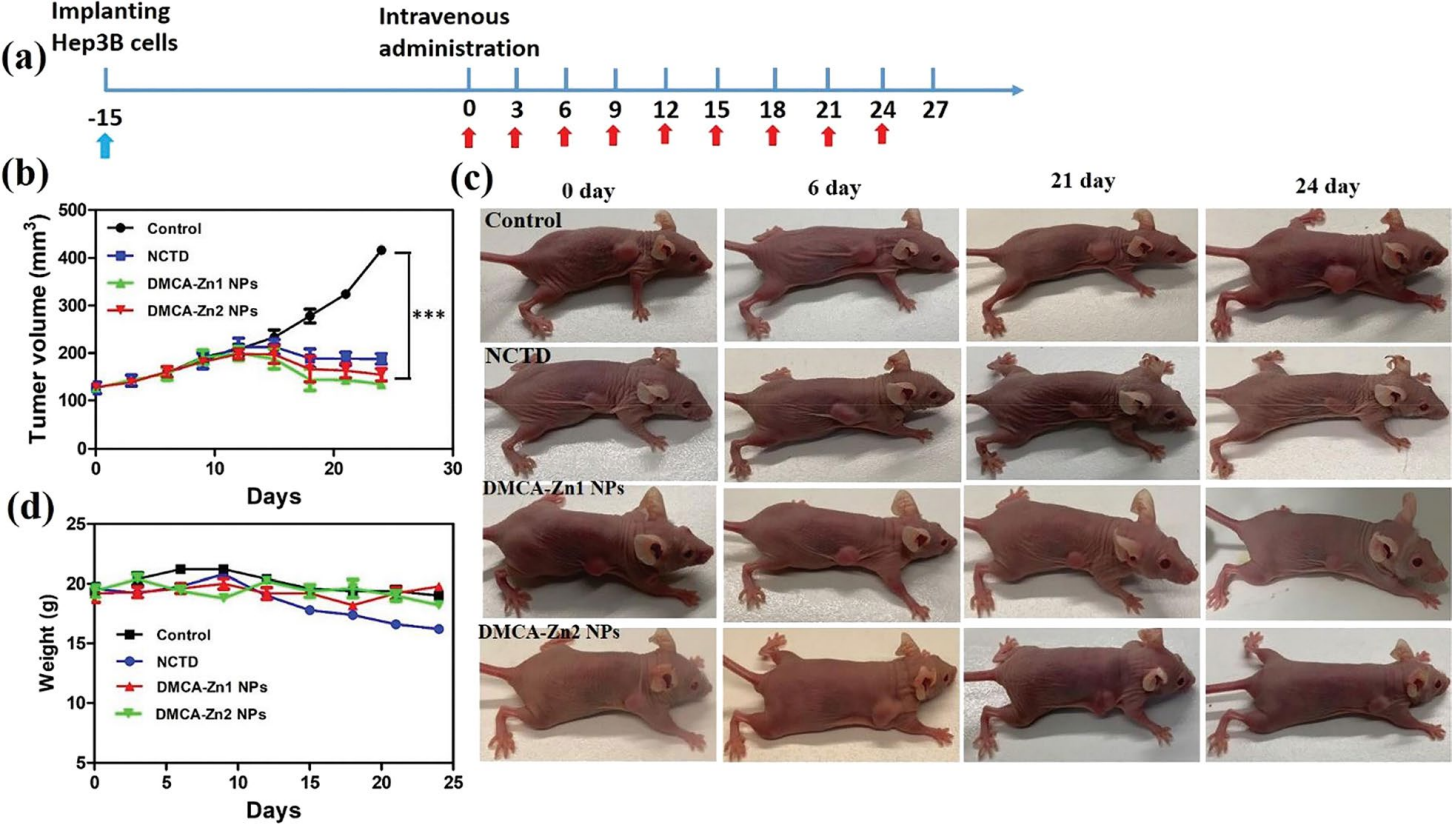
The antitumor efficacy of NCTD, DMCA-Zn1 NPs and DMCA-Zn2 NPs for liver cancer in vivo. **a** The administration process of NCTD, DMCA-Zn1 NPs and DMCA-Zn2 NPs for Hep3B xenograft mice (*n* = 5). **b** The tumor efficacy of NCTD, DMCA-Zn1 NPs and DMCA-Zn2 NPs in Hep3B xenograft mice (*n* = 5) The tumor volumes were normalized to their initial sizes. Statistical significance: *p < 0.05, **p < 0.01 and ***p < 0.001. **c** The photographs of Hep3B xenograft mice treated with NCTD, DMCA-Zn1 NPs and DMCA-Zn2 NPs at 0, 6, 21, and 24 days.** d** The body weights of Hep3B xenograft mice during cancer treatment (*n* = 5). Values are mean ± SD, *n* = 5. ***Values are highly significant difference (*p* < 0.001). Reproduced with permission from reference [87]. Copyright 2022, Royal Society of Chemistry

Zinc is an essential trace element for life. Zinc coordination polymers are also a new type of drug delivery carrier with great application potential. It can lead to the protonation of the coordinate bonds of Zn-based metal-organic coordination polymers in the slightly acidic tumor environment, thereby realizing the targeted drug release [85]. In addition, based on the concentration level of adenosine triphosphate (ATP) in cancer cells is high expression, and the coordination bonding of ATP and Zn^2+^ is much stronger than that of the ligand, Zn-based coordination polymers can further enhance the therapeutic effect on tumor [86]. Wang et al. [87] synthesized two Zn(_II_) coordination polymers [Zn_20_(DMCA)_12_]O_12_ (DMCA = demethylcantharic acid, DMCA-Zn1) and [Zn(DMCA)](H_2_O)_2_ (DMCA-Zn2) from NCTD and Zn(NO_3_)_2_·6H_2_O under solvothermal conditions. Then they transformed DMCA-Zn1 and DMCA-Zn2 crystals into nanoparticles (DMCA-Zn1 NPs and DMCA-Zn2 NPs) by a simple process of mechanical grinding with a biocompatible polymeric surfactant F127, ultrasonic treatment and filtration, with average sizes of around 190 nm and 162 nm. The release rate of DMCA from nanoparticles under slightly acidic conditions (pH = 5.5) is much higher than that under neutral environment, indicating that the two nanoparticles have pH-responsive drug release properties. The two nanoparticles could effectively inhibit the proliferation of HepG2 and Hep3B cells, while they exhibited relatively low toxicity to L927 normal cells. The in vivo studies indicated that they can more effectively suppress Hep3B tumor growth with few side effects compared with free NCTD (Fig. 3). In addition, Zhang et al. [88] prepare a NCTD nanosuspension using hydroxypropyl cellulose-SL and sodium dodecyl sulfate as stabilizers by wet media milling method. The in vitro dissolution of NCTD nanosuspension was 3.27 times that of the micronized NCTD drug at 90 min, indicating that the nanosuspension effectively improved the bioavailability of the free NCTD.

### Microemulsion and self-microemulsion-based NCTD delivery

Microemulsion is defined as a low viscosity, isotropic and thermodynamically stable system composed of oil phase, water phase, surfactant and cosurfactant in appropriate proportions [89]. It can improve the solubility and bioavailability of the free NCTD, and enhance the targeting effect of the drug in vivo. Zhang et al. [90] prepared a NCTD-loaded W/O microemulsion, with an average size of (44.5 ± 8.6) nm. Compared with NCTD injection, the elimination t_1/2_, mean residence time (MRT) and area under the curve (AUC) of NCTD microemulsion were increased by 2.62, 1.3 and 3.2 times, respectively, indicated that NCTD-loaded microemulsion had relatively longer circulating time in vivo. Moreover, the overall drug targeting efficiency of liver was enhanced from 3.66% to 6.10%. The microemulsion vehicles also decreased the kidney distribution of NCTD.

Self-microemulsion, an isotropic mixture of surfactants, co-surfactants, oil and drugs, can spontaneously form O/W nanoemulsion ranging 1-100 nm upon contact with aqueous medium under the digestive movement of the stomach or upper small intestine. It has the advantages of easy preparation, good stability, and high bioavailability [91]. Zeng et al. [92] prepared a NCTD-loaded self-nanoemulsifying drug delivery system (NCTD-SNEDDS) containing 50% Ethyl Oleate, 35% Cremophor EL, 15% ethylene glycol and 10 mg NCTD. The optimized SNEDDS had a size of 36.31 nm and a polydispersity index (PDI) of 0.05, which could withstand extensive dilution and exhibit a sustained drug release property, thereby improving the bioavailability of NCTD. Gui et al. [93] also prepared a NCTD solid self-microemulsion by spherulite technology one-step curing method. The particle size of NCTD solid self-microemulsion was 22.76 nm, the zeta potential was − 2.77 mV, the average EE and yield was 77.39% and 84.5%, respectively, and the self-emulsification could be completed within 50 s.

### Polymer-conjugated NCTD delivery

Based on the EPR effect of macromolecular drugs, conjugation of drugs to suitable macromolecules is considered to be a useful approach to prolong drug retention at target sites or to deliver drugs to target sites. Based on the repeating structure of (1,4)-linked-2-amino-2-deoxy-β-D-glucan of CS, CS or CS derivatives-anticancer drug conjugates are used as drug delivery carriers received widespread attention [94]. Several studies have shown that the anticancer drug NCTD conjugated with CS or CS derivatives can significantly improve the water solubility and in vivo pharmacokinetics of NCTD, and passively accumulate NCTD into tumor tissues, thereby greatly enhancing the antitumor effect and reducing the toxicity of NCTD. Xu et al. [94] synthesized NCTD-conjugated CS conjugates (NCTD-CSs) with different degrees of substitution (DS, 60.2% and 97.9%). Compared with CS, the conjugates had better water solubility and exhibited sustained drug release behavior, releasing less than 6% NCTD from the conjugates within 16 days via the hydrolysis of ester bonds in PBS (pH 5.0 and 7.4). The NCTD-CSs could arrest MGC80-3 cell cycle at G2/M phase and induce cell apoptosis similarly to NCTD. Moreover, Li et al. [95] also synthesized a NCTD-CS via alcoholysis reaction. Due to the EPR effect, NCTD-CS displayed higher tumor inhibition rate (45.82 ± 12.12) % than that of free NCTD (35.87 ± 6.25) % in EMT6 breast cancer model.

The application of CS was often hampered largely by its solubility only in acidic environments with pH values lower than 6 [96]. Hydroxypropyltrimethyl ammonium chloride chitosan (HACC) is a partially quaternized CS derivative that could be soluble over the entire pH range, and its quaternized cationic nature provides strong electrostatic interaction with negatively charged tumor cells [97]. Xu et al. [97] synthesized NCTD-conjugated HACCs (NCTD-HACCs) (DS, 12.2% and 24.8%) with good water solubility. NCTD was released from the NCTD-HACCs via hydrolysis, which was faster at pH 5.0 than at pH 7.4, showing a biphasic drug release pattern with an initial fast release followed by a slow release. Compared with free NCTD, the NCTD-HACCs showed higher tumor growth inhibition in S180 tumor-bearing mice due to the EPR effect.

Carboxymethyl chitosan (CMCS) is a kind of carboxymethylated CS derivative. Due to its excellent water solubility, and the presence of functional groups (amino and carboxyl groups), CMCS has become a promising carrier for conjugating the hydrophobic drugs such as NCTD to improve the therapeutic efficiency [98]. Jiang et al. [99] synthesized a NCTD-conjugated CMCS (CMCS-NCTD), which had a good anti-hepatocellular carcinoma effect and a better ameliorating effect on liver damage caused by tumor cells than NCTD. CMCS-NCTD significantly increased the levels of tumor necrosis factor-α (TNF-α), interferon-γ (IFN-γ), tissue inhibitor of matrix metalloproteinase (TIMP)-1 and E-cadherin, and reduced the levels of alanine aminotransferase (ALT), aspartate aminotransferase (AST), vascular endothelial growth factor (VEGF) and MMP-9, indicating that CMCS-NCTD may prevent tumor growth by regulating key cytokines associated with tumor immunity, angiogenesis, extracellular matrix degradation and epithelial mesenchymal transition. Furthermore, CMCS-NCTD could protect liver from oxidative damage causing by tumor via enhancing the levels of superoxide dismutase (SOD) and glutathione peroxidase (GSH-Px). Chi et al. [98] also prepared a NCTD-conjugated CMCS conjugate (CNC), which exerted enhanced inhibitory effects and reduced systemic toxicity in H22 hepatocellular carcinoma model compared with free NCTD. Additionally, CNC could enhance immune responses through regulating the expressions of TNF-α and IFN-γ. Moreover, CNC displayed strong hepatic tropism with Re value of 1.438, and decreased distribution in the heart and kidneys compared to NCTD, thereby displaying reduced toxicity to these organs. CMCS-NCTD could also exert anti-metastasis effects by inhibiting tumor angiogenesis and decreasing degradation of extracellular matrix by regulating the levels of VEGF, MMP-9 and TIMP-1 in Lewis lung carcinoma metastasis model [100]. Besides, the conjugate showed a high antitumor effect in SGC-7901 tumor model with a tumor inhibition rate of 59.57%, and which might be mediated by increasing the levles of TNF-α, Bax and Caspase-3 and reducing the levels of CD34, VEGF, MMP-2, MMP-9 and Bcl-2 [101]. Overall, CNC conjugate based on CMCS as polymer carriers might be used as a potential clinical alternative for NCTD in cancer therapy.

**Fig. 4:**
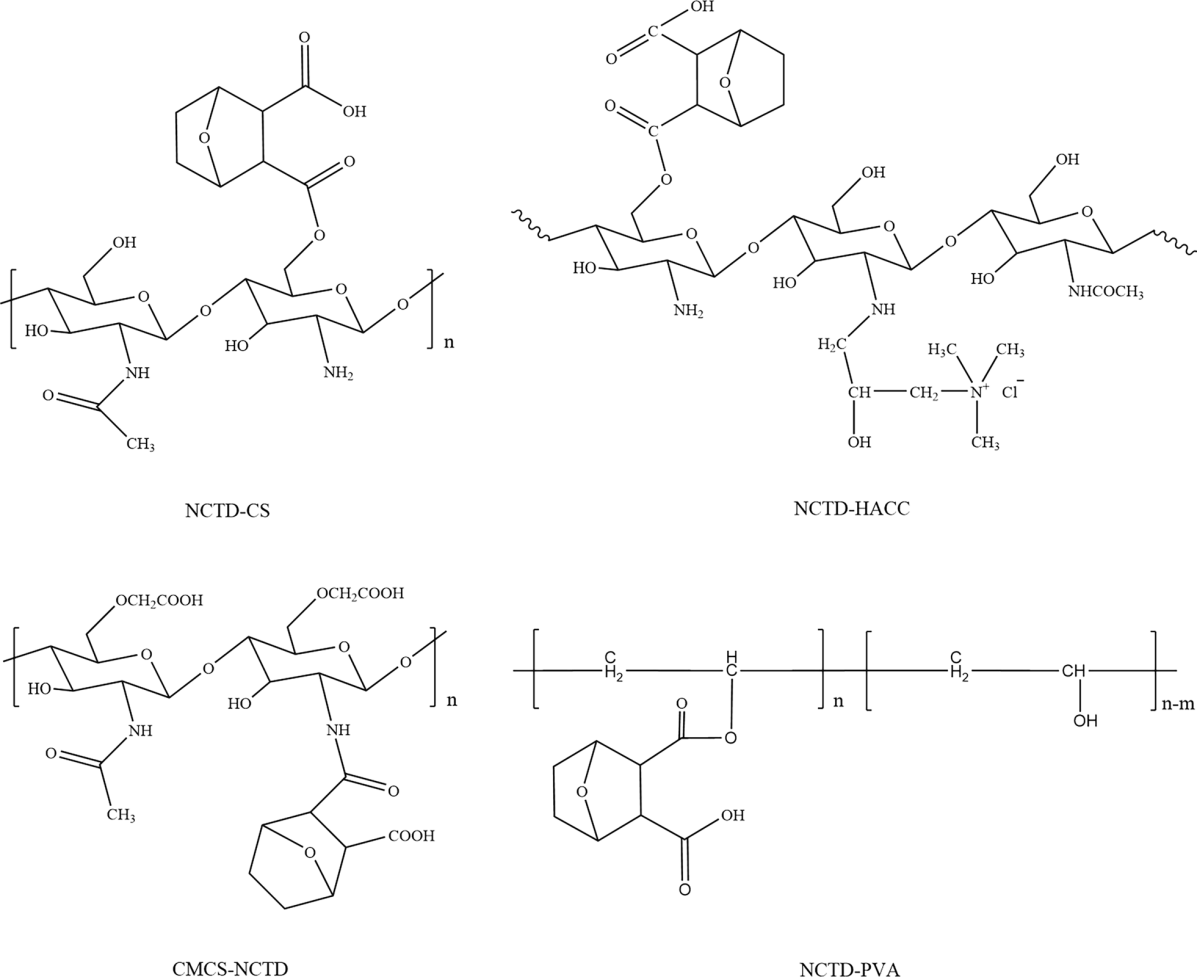
The structure of polymer-conjugated NCTD

Besides, poly(vinyl alcohol) (PVA), a high molecular weight polymer with multiple hydroxyl groups and good biocompatibility, can easily be conjugated with NCTD to increase the accumulated drug amount in the tumor tissue by the EPR effect, thus enhance the drug delivery efficiency. Li et al. [95] synthesized a NCTD-PVA via alcoholysis reaction. NCTD was released from the conjugates via hydrolysis, faster in PBS (pH 5.0) than that in PBS (pH 7.4). NCTD-PVA could inhibit human esophageal carcinoma ECA-109 cell and murine breast cancer EMT6 cell growth in a dose-dependent manner. NCTD-PVA could also induce ECA-109 cell apoptosis and arrested cell cycle at the S phase, activate caspase-3/-8. In the EMT6 tumor-bearing mouse model, NCTD-PVA displayed higher tumor inhibition rate [(56.17 ± 11.34) %] than that of free NCTD [(35.87 ± 6.25) %]. The structure of four polymer-conjugated NCTD are showed in Fig. 4.

### Microsphere-based NCTD delivery

Lipid microspheres refer to a particle dispersion system with an average particle size of less than 200 nm, which is prepared by dissolving drugs in fatty oil, and then emulsified and dispersed in water phase by phospholipids. Lipid microspheres have the advantages of increasing the solubility of poorly soluble drugs, reducing drug irritation and toxic side effects, sustained and slow release of drugs, prolonging the half-life of drugs in the body, and improving drug bioavailability and targeting ability. They are physically stable, biodegradable, biocompatible, and easy to prepare [102]. Consequently, lipid microspheres are ideal carriers for NCTD. Wang et al. [45] prepared a NCTD-loaded lipid microsphere by high-pressure homogenization process and localizing the drug at the interfacial surface of the oil and aqueous phases. NCTD-loaded lipid microsphere with over 80% NCTD loaded in the interfacial surface were stable for 2 months, and were suitable for *i.v.* injection with less pain and irritation. Lin et al. [103] prepared a NCTD-loaded lipid microsphere by homogenization method. Compared with NCTD injection, the microsphere significantly reduced the cardiac and renal toxicity in A549, BEL7402 and BCAP-37 tumor model. Moreover, The LD_50_ value of NCTD injection for female mice and male mice administered *i.v.* was 10.10 and 8.93 mg/kg, respectively; while the LD_50_ of NCTD-loaded lipid microsphere was 15.67 mg/kg for female mice and 16.64 mg/kg for male mice, which was twice higher than that of NCTD injection. Moreover, Ma et al. [104] prepared a NCTD-phospholipid complex (NPC)-loaded lipid microsphere (NPCLM). The NPC was firstly produced to increase the lipophilic properties of NCTD and a concentrated homogenization method was then used to prepared the NPCLM. The lipophilicity of NPC was significantly increased almost 224-fold compared with NCTD. After optimizing the emulsification process, the EE was significantly increased from 21.6 to 84.6%, and a highly sterilization stability was achieved with only a small change in particle size from (168.2 ± 39.4) nm to (173.4 ± 43.5) nm. NPCLM had slow drug release properties, only releasing 4.68% and 14.21% of NCTD within 15 min and 4 h, respectively. Furthermore, NPCLM showed an increased accumulation of NCTD in the liver, spleen and lung, which were 1.67, 1.49 and 1.06 times higher than in the injection group, while the content of NCTD was reduced 0.96-fold in the kidney. Therefore, this NPCLM increased liver targeting and reduced renal toxicity of NCTD.

Human serum albumin (HSA) is a natural drug carrier of the human body, which can increase the solubility of poorly soluble drugs in plasma and reduce the toxicity of drugs. As a material for pharmaceutical preparations, it has the characteristics of high compatibility and low reactogenicity [105]. The preparation of NCTD-loaded microspheres with HSA as a carrier can not only increase the solubility of the drug, but also protect the drug from the external environment and improve the stability of the drug. The NCTD encapsulated in the microspheres have a sustained-release effect, which can reduce the toxicity and side effects while prolonging the therapeutic efficacy. Yan et al. [106] prepared a NCTD encapsulated albumin microspheres by the emulsion crosslinking method. The microspheres had a good sustained-release efficacy, and significantly prolonged the drug circulation time, and had higher AUC inside liver than the NCTD injection with a target index of 3.49. Moreover, no histological change occurred in the rat liver. Wang et al. [107] also prepared a NCTD-loaded CS microsphere by emulsification cross-linking process with liquid paraffin as oil phase, Span-80 as emulsifier, and formaldehyde as cross-linking agent. The CS microsphere showed a sustained drug release property compared with free NCTD.

### Liposome and emulsion hybrid delivery system-based NCTD delivery

In order to further improve EE, increase sterilization stability, and enhance antitumor effect of NCTD-loaded drug delivery systems, some researchers have also proposed that the delivery of NCTD in a liposome-emulsion-hybrid (LEH) nanoparticle carrier (NLEH), which encapsulates the emulsions into liposomes. Phospholipid complexes methods were used for increasing the lipophilicity of NCTD, then NCTD phospholipid complexes were not only loaded in the oil phase and oil-water interface surface of emulsions, but also encapsulated in phospholipid bilayers. NLEH has good size distribution, with a particle size of (163.8 ± 1.082) nm (PDI < 0.084), and exhibited an improved EE (89.3%) and an excellent sterilization stability. Compared with NCTD liposomes and NCTD emulsions, NLEH had a better antitumor effect by promoting absorption (1.93-fold), extending circulation time (2.08-fold), improving tumor targeting ability (1.19 times) and tumor penetration, and enhancing antitumor immune effect. Moreover, NLEH decreased the targeting efficiency in the heart and kidneys, and achieved a better biosafety [108]. Therefore, the liposome and emulsion hybrid delivery systems are potential carriers for NCTD delivery in the treatment of hepatocellular carcinoma (HCC).

## Active targeted drug delivery systems

Actively targeted drug delivery systems mainly use some specific or highly expressed biomarkers at the tumor site to deliver drugs to specific cells in a targeted manner. The surface of the drug delivery system is modified with specific ligands, such as proteins, antibodies, polypeptides or small chemical molecules, which can specifically bind to highly expressed receptors or antigens on the surface of the cell membrane, triggering endocytosis, thereby achieving drug delivery [109]. Based on some antigens or receptors that are highly expressed on the surface of tumor cells, such as CD19, carbonic anhydrase IX (CA IX), glycyrrhetinic acid (GA) receptors, asialoglycoprotein receptor (ASGPR), integrin α5 (ITGA5) receptor and folate acid (FA) receptor, some researchers have designed and constructed a variety of NCTD-loaded actively targeted nano-drug delivery systems to increase the concentration of NCTD in target cells, thereby enhancing its efficacy and reducing its toxicity and side effects (Table 3).

**Table 3:** Active targeted drug delivery systems of norcantharidin

Drug delivery system	Ligand/antibody	Receptor/antigen	Composition	Efficacy	Ref., year
2E8-NCTD-liposomes	A murine anti-human CD19 monoclonal antibody 2E8	CD19	SPC/CHO/mPEG_2000_-PE/Mal-PEG_2000_-DSPE (molar ratio of 2: 1: 0.08: 0.02), 2E8-Mal-PEG_2000_-DSPE	Targeting efficiency: Nalm-6, Raj cells (CD19^+^): 2E8-NCTD-liposomes > NCTD-liposomes; Molt-3, K562 cells (CD19^–^): 2E8-NCTD-liposomes ≈ NCTD-liposomes; cell viability (10–50 µmol/L 2E8-NCTD-liposomes): Nalm-6 cells < Molt-3 cells; cell viability (Nalm-6 cells): 2E8-NCTD-liposomes < NCTD-liposomes < free NCTD	[111], 2010
Hm2E8b-NCTD-liposomes	A humanized anti-human CD19 monoclonal antibody, Hm2E8b	CD19	SPC/CHO/mPEG_2000_-PE/Mal-PEG_2000_-DSPE (molar ratio of 2: 1: 0.08: 0.02), Hm2E8b-Mal-PEG2000-DSPE	Targeting efficiency: HAL-01 cells (CD19^+^): Hm2E8b -NCTD-liposomes > NCTD-liposomes; Molt-3 cells (CD19^–^): Hm2E8b-NCTD-liposomes ≈ NCTD-liposomes; inhibition rate (2E8-NCTD-liposomes): HAL-01 cells > Molt-3 cells; inhibition rate (HAL-01 cells): Hm2E8b-NCTD-liposomes > NCTD-liposomes > free NCTD; cell apoptosis ↑; HLF-SLUG, NFIL3, c-myc, p-STAT5, STAT5 ↓, caspase-3/-6/-7/-9, P53, p-P53 ↑	[112], 2018
Anti-carbonic anhydrase IX (CA IX) NCTD nano-micelle	An anti-CA IX monoclonal antibody	CA IX	NCTD, DSPE-PEG2000-Mal, DTT, EDTA, anti-CA IX	A549 cell viability: anti-CA IX NCTD nano-micelle < NCTD nano-micelle < NCTD; A549 tumor inhibition rate (*iv.*, once daily, 8 days): NCTD (1.0 mg/kg) = 54.78%, NCTD nano-micelle (0.5, 1.0 mg/kg) = 45.22%, 64.35%, anti-CA IX NCTD nano-micelle (0.5, 1.0 mg/kg) = 67.82%, 75.65%	[115], 2017
NCTDloaded liposome modified with stearyl glycyrrhetinate (SG) (SG-NCTD-LIP)	SG	Glycyrrhetinic acid (GA) receptor	1: 5 NCTD‑phospholipid mass ratio, EPC (0.4%), 1: 7 cholesterol-phospholipid mass ratio, SG (0.04%), 2 mL absolute ethanol, 15 mL PBS (pH 7.0)	HepG2 cells: SG‑NCTD‑LIP (IC_50_ = 16.93 µg/mL) < NCTD‑LIP (IC_50_ = 24.03 µg/mL) < free NCTD (IC_50_ = 49.79 µg/mL)	[117], 2018
mPEG-PCL-PEI-GA (MPG)/NCTD nanoparticles (AT NPs)	GA	GA receptor	NCTD (6–20 mg), MPG copolymer (94−80 mg), 1 mL DMSO, 1 mL methyl alcohol, 10 mL deionized water	HepG2 cells: cell viability: free NCTD > NAT NPs > AT NPs; cellular uptake ↑; H22 tumor inhibition rate and median survival time (*iv.*, 2.5 mg/kg, every 2 days, 4 times): free NCTD (44 days) < NAT NPs (56 days) < AT NPs (68 days); tumor/muscle (T/M) ratio: free NCTD > NAT NPs > AT NPs; cell apoptosis and cell cycle (G2 and S phase): free NCTD < NAT NPs < AT NPs; Ki-67, microvessel density (MVD): free NCTD > NAT NPs > AT NPs; CD31: free NCTD < NAT NPs < AT NPs	[118], 2018
NCTD-loaded liposome modified with GA and (trans-activator of transcription, TAT)	GA and TAT	GA receptor	NCTD, lecithin, cholesterol, DSPE-PEG5000-GA, DSPE-PEG2000-TAT	HepG2 cells: cell viability: NCTD-loaded liposome < NCTD solution	[119], 2020
Lac-NCTD-NPs	Lactosyl	Asialoglycoprotein receptor (ASGPR)	0.1 g CS, 0.2% aqueous acetic acid solution, 0.1 g Lac-NCTD, 21 mL 1.2 g/L TPP water solution	HepG2, SMMC-7721 cells: IC_50_ (48 h): Lac-NCTD-NPs < Lac-NCTD; H22 tumor inhibition rate (*ip.*, once daily, 8 days): Lac-NCTD (3.3, 6.6, 13.8 mg/kg) = 27.1%, 41.7%, 53.5% < Lac-NCTD-NPs (3.3, 6.6, 13.8 mg/kg) = 31.9%, 63.9%, 70.1%; thymus and spleen index: Lac-NCTD < Lac-NCTD-NPs	[122, 123], 2009, 2010
Lactosyl-NCTD N-Trimethyl chitosan (TMC) nanoparticles (Lac-NCTD-TMC-NPs)	Lactosyl	ASGPR	0.15 g TMC, 50 mL distilled water containing 0.03 g Lac-NCTD, 25 mL 1.2 g/L TPP water solution	HepG2 cells: IC_50_: Lac-NCTD-TMC-NPs < Lac-NCTD-CS-NPs < Lac-NCTD; induce cell apoptosis; Cellular uptake (HepG2 cells) ↑; H22 tumor inhibition rate (*ip.*, once daily, 9 days): NCTD (2.0 mg/kg) = 31.2% < Lac-NCTD (6.6 mg/kg) = 38.3% < Lac-NCTD-CS-NPs (6.6 mg/kg) = 51.06% < Lac-NCTD-TMC-NPs (6.6 mg/kg) = 69.5%; spleen coefficient and thymus coefficient ↑	[96], 2012
NCTD-associated galactosylated chitosan (GC) nanoparticle (NCTD-GC-NPs)	GC	ASGPR	100 mg GC, 50 mL 0.2% aqueous acetic acid solution, 20 mg NCTD, 1.2 g/L TPP water solution	Cellular uptake (HepG2, SMMC-7721 cells) ↑; in vitro cytotoxicity (HepG2, SMMC-7721 cells): NCTD < NCTD-CS-NPs < NCTD-GC-NPs	[124], 2009
NCTD-GC-NPs	GC	ASGPR	GC, 0.2% aqueous acetic acid solution, NCTD, 1.2 g/L TPP water solution	Cellular uptake (Bel-7402, HL-7702 cells) ↑; in vitro cytotoxicity (Bel-7402, HL-7702 cells): NCTD < NCTD-CS-NPs < NCTD-GC-NPs; H22 tumor inhibition rate (*ip.*, once daily, 8 days): NCTD (2.0 mg/kg) = 28.97% < NCTD-CS-NPs (0.5, 2.0, 4.0 mg/kg) = 25.71%, 37.86%, 56.87% < NCTD-GC-NPs (0.5, 2.0, 4.0 mg/kg) = 26.42%, 43.56%, 59.52%	[125], 2010
Gal-GAOStNC-LP	Stearin glycyrrhetinic acid ester-3-O-galactosidase, Gal-GAOSt	Galactose receptor	NCTD, soy lecithin, cholesterol, sodium ursodeoxycholate, Gal-GAOSt	Targeting index: liver (5.213 ± 1.320) > 1, spleen (1.980 ± 1.375) > 1, heart (0.496 ± 0.837) < 1, lung (0.871 ± 0.659) < 1, kidney (0.468 ± 0.914) < 1	[126], 2009
Galactose-cholesterol modified NCTD liposomes (Gal-NCTD-Lips)	Galactose	ASGPR	Phospholipid/galactosylated cholesterol = 3: 1 (weight ratio), lipid/drug = 12: 1 (weight ratio)	-	[127], 2019
Arabinogalactan-anchored polymeric micelles of NCTD (NCTD-M)	Arabinogalactan (AG)	ASGPR	N-(4-methylimidazole)-hydroxyethyl-chitosan (MHC): AG = 1: 3 (mass ratio), 5 mg NCTD	A significant liver-targeting effect; HepG2 cells: enhance cellular uptake of NCTD, promote the lysosomal escape, inhibit cell invasion and induce cell apoptosis; H22 tumor inhibition rate (*iv.*, once every 3 days, 15 days): NCTD (2.0 mg/kg) < NCTD-M (2.0 mg/kg)	[128], 2018
NCTD/Galactosamine- hyaluronic acid-Vitamin E succinate micelles (NCTD/Gal-HA-VES micelles)	Gal, HA	CD44, ASGPR	20 mg Gal-HA-VES, NCTD	Cellular uptake (HepG2 and MCF-7 cells) ↑; inhibit P-gp expression (MCF-7/Adr cells); IC_50_ (HepG2 and MCF-7 cells, MCF-7/Adr cells): NCTD/Gal-HA-VES < NCTD/HA-VES < NCTD; cell apoptosis ↑; the micelles accumulated in liver, spleen, tumors ↑ and in kidneys ↓; HepG2 tumor inhibition rate (*iv.*, every 3 days for 6 times, 18 days, 10 mg/kg): NCTD = (44.01 ± 5.78) % < NCTD/HA-VES = (68.74 ± 2.72) % < NCTD/Gal-HA-VES = (77.87 ± 3.36) %	[129], 2018
NCTD-loaded RGD-lipid-polymer hybrid (LPH) nanoparticles (RGD-LPH-NCTD)	RGD (Arg-Gly-Asp)	Integrin α5 (ITGA5) receptor	Lecithin (2 mg), PEG-DSPE (18 mg), RGD-PEG-DSPE (2 mg), NCTD (1.25 mg), PEI_10 K_ (375 µg), PLGA (1.875 mg)	Cellular uptake (LM2 cells) ↑; LM2, MDA-MB-231, SUM159 cells: cell viability: free NCTD > LPH-NCTD > RGD-LPH-NCTD; reduce colony formation ↑; inhibit cancer stem cell-like property ↑; reduce both active (non-phospho-β-catenin) and total β-catenin protein levels; the targeting capability in primary mammary tumor and metastatic lung tumor ↑; inhibit LM2 tumor growth and lung metastasis (*iv.*, 3 times a week for 5 consecutive weeks), reduce β-catenin level and its nuclear localization, increase E-cadherin expression	[135], 2019
Folate acid (FA)-conjugated NCTD-loaded stealth niosomes	FA	FA receptor	FA-PEG-chol, F127-chol, Span-80, 3.8 mg NCTD, 0.15 mL absolute ethanol, 0.05 mL ethyl acetate, 5 mL PBS	Hela cells: cellular uptake ↑; IC_50_ (12 h): FA-conjugated NCTD-loaded stealth niosomes (46 µg/mL) < FA + FA-conjugated NCTD-loaded stealth niosomes (91 µg/mL) < NCTD-loaded stealth niosomes (148 µg/mL) < NCTD (261 µg/mL)	[137], 2013
Diacid metabolite (DM)-NCTD-loaded, FA-modified, polyethylene glycolated (DM-NCTD/FA-PEG) liposomes	FA	FA receptor	DSPC, cholesterol, DSPE-PEG2000, DSPE-PEG2000-FA = 2: 1: 0.11: 0.017 (molar ratio), DM-NCTD	H22 cells: IC_50_ (48 h): DM-NCTD (30.0 ± 1.73) µg/mL < DM-NCTD/FA-PEG liposomes (50.1 ± 1.04) µg/mL < DM-NCTD/PEG liposomes (92.5 ± 1.31) µg/mL; tumor-targeting efficiency: DM-NCTD/PEG liposomes [relative intake rate 4.86, tissue/tumor-targeting efficacy 12.81%, relative targeting efficiency 2.36, peak concentration ratio 4.78] < DM-NCTD/FA-PEG liposomes (9.25, 24.44%, 4.50, and 9.24); H22 tumor inhibition rate (*iv.*, once daily, 9 days, 2.0 mg/kg): DM-NCTD = 30.14% < DM-NCTD/PEG liposomes = 40.41% < DM-NCTD/FA-PEG liposomes = 67.81%; tumor-cell apoptosis ↑	[138], 2016
FA receptor-targeted NCTD/tetrandrine (Tet) dual-drug loaded lipid nanoparticles [(FA-LP@Tet/(MSNs@NCTD)]	FA	FA receptor	FA-DSPE-PEG2000 (0.18 mg), DSPE-PEG2000 (0.72 mg), DSPC (6.3 mg), cholesterol (2.8 mg), Tet (2 mg), 5 mL dichloro- methane, 10 mL anhydrous ethanol, PBS buffer (10 mL, pH 7.4) containing 2 mg of MSNs@NCTD	HepG2 cells: cellular uptake ↑; IC_50_ (HepG2, HepG2/Adr, MCF-7, LO2 cells): FA-LP@Tet/(MSNs@NCTD) < LP@Tet/(MSNs@NCTD) < LP/(MSNs@NCTD) < NCTD; induce cell apoptosis: HepG2 cells < HepG2/Adr cells; inhibit P-gp expression	[182], 2019
FA-LB(ABT-737)-(DM-NCTD@CHMSN)	FA	FA receptor	DSPC, cholesterol, DSPE-PEG2000, DSPE-PEG2000-FA = 2: 1: 0.11: 0.017 (molar ratio), CHMSN/DM-NCTD = 2.5: 1 (weight ratio), ABT-737/ DM-NCTD = 1: 10 (mol ratio), DM-NCTD@CHMSN/lipid = 0.02: 1 (weight ratio)	In vitro cytotoxicity, cell apoptosis (H22 cells): DM-NCTD < ABT-737 < DM-NCTD + ABT-737 < LB(ABT-737)-(DM-NCTD@CHMSN) < FA-LB(ABT-737)-(DM-NCTD@CHMSN); mitochondrial membrane potential ↓; cellular uptake ↑; H22 tumor inhibition rate (14 days): ABT-737 (*ip.*, 50 mg/kg) = 21.0% < DM-NCTD (*iv.*, 2 mg/kg) = 34.6% < DM-NCTD (*iv.*, 2 mg/kg) + ABT-737 (*ip.*, 50 mg/kg) = 45.8% < FA-LB(ABT-737)-(DM-NCTD@CHMSN) (*iv.*, 2 mg/kg) = 69.6%; tumor-cell apoptosis and Cytochrome C expression ↑	[184], 2020

### Monoclonal antibody-based active targeted drug delivery

#### Anti-CD19 monoclonal antibody-based NCTD delivery

CD19 is highly expressed on B-lineage leukemia stem cells (B-LSCs) and their progeny, but not on the normal hematopoietic stem cells, granulocytes and platelets [110]. Based on this, Zhang et al. [111] prepared a NCTD-encapsulated liposome modified with a murine anti-human CD19 monoclonal antibody 2E8 (2E8-NCTD-liposomes) by using post-incorporation technology, with average size of 118.32 nm and average EE of 46.51%, which can specifically target CD19^+^ leukemia cells for the treatment of B lineage hematologic malignancies. Moreover, the immunoliposomes were able to specifically target the B-LSCs and their progeny by inducing B-LSCs apoptosis by downregulating the HLF and upregulating the NFIL3 (nuclear factor, IL3 regulated) expressions. They also prepared NCTD-encapsulated liposomes modified with a novel humanized anti-human CD19 monoclonal antibody, Hm2E8b (Hm2E8b-NCTD-liposomes) that specifically target the B-LSC-related E2A-HLF/SLUG axis against B-LSCs. This liposome reduced HLF protein levels and induced HAL-01 cell apoptosis by regulating the expression of SLUG, nuclear factor interleukin-3 (NFIL3) and c-myc, thereby inducing p53 and mitochondrial caspase cascades [112].

#### Anti-CA IX monoclonal antibody-based NCTD delivery

CA IX is a transmembrane protein that belongs to the carbonic anhydrase family. CAIX is lowly expressed in normal human tissues, but highly expressed in lung cancer and other malignant tumor tissues, and plays an important role in the growth, infiltration and metastasis of tumor cells [113, 114]. Wang et al. [115] prepared an anti-CA IX NCTD nano-micelle with drug loading ability, EE, size and zeta potential of (1.26 ± 0.03) %, (80.93 ± 1.01) %, (146.5 ± 48.9) nm and − (14.79 ± 0.67) mV, respectively. Compared with NCTD nano-micelle, anti-CA IX NCTD nano-micelle specifically binded to the highly expressed CA IX antigen at the tumor site, which can not only inhibit the catalytic activity of CA IX to prevent tumor deterioration, but also guide drug-loaded micelles to the tumor tissue, thus exhibiting enhanced antitumor effect on A549 cells and A549 tumor-bearing mice, with a tumor inhibition rate of 75.67% in vivo.

### Receptor-based active targeted drug delivery

#### GA receptors-based NCTD delivery

The GA receptors are expression-rich on the liver cell membrane. By targeting these receptors, drugs can be specifically delivered into liver cancer sites, thereby reducing the accumulation of drugs in normal tissues [116]. Zhu et al. [117] prepared a NCTDloaded liposome modified with stearyl glycyrrhetinate (SG) (SG-NCTD-LIP), which showed an enhanced antitumor effect (1.42-fold) by the interaction between SG and GA receptors on the cell membrane compared with NCTD-LIP. Zhang et al. [118] designed a GA-conjugated NCTD-loaded polymeric nanoparticles (AT NPs), which had higher targeting ability on HepG2 cells and increased cell apoptosis and enhanced the G2 and S phase arrest compared to non-conjugated nanoparticles (NAT NPs). In vivo anti-tumor evaluation indicated that the AT NPs significantly inhibited tumor growth, prolonged survival of tumor-bearing mice, and decreased microvessel density (MVD). Chang et al. [119] constructed a NCTD-loaded liposome modified with GA and (trans-activator of transcription, TAT). This dual-targeted liposome has good sustained-release properties, and its inhibitory effect on HepG2 cells was 2.14 times higher than that of NCTD.

#### ASGPR-based NCTD delivery

ASGPR, also known as the “Ashwell-Morell receptor”, was the first cellular mammalian lectin discovered by Ashwell and Morell when they were studying the metabolism of mammalian plasma glycoproteins [120]. ASGPR is a receptor expressed mainly on the surface of liver sinusoidal and basolateral cells. It can exclusively recognize, bind and clear desialylated glycoproteins with exposed non-reducing D-galactose (Gal) or N-acetylgalactosamine (GalNAc) as end groups [121]. NCTD is a commonly used drug for the treatment of liver cancer in clinical. Therefore, ASGPR-mediated targeted drug delivery systems combined with NCTD for liver cancer therapy has drawn extensive attention.

Hu et al. [122] prepared an active liver-targeting CS nanoparticles (Lac-NCTD-NPs) by ionic cross-linkage process using synthesized lactosyl-NCTD (Lac-NCTD) as antitumor drug. Compared with free NCTD and Lac-NCTD, Lac-NCTD-NPs can significantly prolong the action time of drugs and enhanced the antitumor effect of ASGPR-expressed HepG2 and SMMC-7721 cells. Moreover, the tumor inhibition rate of Lac-NCTD-NPs was 63.9% on H22 tumor model, which was significantly higher than that of Lac-NCTD (41.7%) at the dose of 6.6 mg/kg. Lac-NCTD-NPs can also significantly increase the thymus and spleen indices of nude mice, indicating that they have the function of protecting organs and improving immunity [123]. Guan et al. [96] also prepared lactosyl-NCTD N-Trimethyl chitosan (TMC) nanoparticles (Lac-NCTD-TMC-NPs). Compared with Lac-NCTD and Lac-NCTD CS NPs (Lac-NCTD-CS-NPs), Lac-NCTD-TMC-NPs had the strongest antitumor effect both on the HepG2 cell and the murine hepatocarcinoma 22 tumor models, indicating that the recognition of ASGPR located on the surface of hepatoma cells can enhance the liver targeting ability.

Wang et al. [124] prepared NCTD-associated galactosylated CS nanoparticles (NCTD-GC-NPs) using galactosylated CS as carrier. The nanoparticles have significant slow drug release properties and pH-sensitive release properties that followed Higuchi equation. Compared with NCTD-loaded CS nanoparticles (NCTD-CS-NPs), NCTD-GC-NPs showed stronger cytotoxicity and compatibility with SMMC-7721 and HepG2 cells. Hu et al. [125] also prepared a NCTD-GC-NPs. The IC_50_ values of NCTD, NCTD-CS-NPs and NCTD-GC-NPs were (18.84 ± 1.87), (16.38 ± 1.48), (7.12 ± 1.94) µg/mL for Bel-7402 cells. The inhibition ratios of 2.0 mg/kg NCTD, NCTD-CS-NPs, NCTD-GC-NPs on mice bearing H22 liver tumor were 28.97%, 37.86% and 43.56%. Therefore, NCTD-GC-NPs had stronger antitumor activity than NCTD and NCTD-CS-NPs by targeting ASGPR. Wu et al. [126] prepared a glycyrrhetinic acid derivatives (stearin glycyrrhetinic acid ester-3-O-galactosidase, Gal-GAOSt) modified NCTD liposome (Gal-GAOStNC-LP) by thin film dispersion method. The liver targeting index of Gal-GAOStNC-LP reached 5.213, indicating the liposome has obvious liver targeting ability by the interaction of Gal and ASGPR. Zhou et al. [127] also synthesized galactose-cholesterol conjugates using galactose and cholesterol chloroformate as substrates. Then, they prepared galactose-cholesterol modified NCTD liposomes (Gal-NCTD-Lips) by thin-film ultrasonic dispersion method. In vitro release results showed that the liposomes had good sustained-release characteristics compared with NCTD solution.

Zhang et al. [128] prepared NCTD-loaded polymer micelles by conjugating arabinogalactan (AG) on the surface of N-(4-methylimidazole)-hydroxyethyl-chitosan (MHC) (NCTD-M). The micelles have a significant liver-targeting effect through the specific recognition of AG and ASGPR, which resulted in higher cytotoxicity and cell apoptosis rate, and stronger ability to inhibit cell invasion than that of free NCTD, and in vivo study results also supported this conclusion. Jiang et al. [129] designed a multifunctional self-assembled micelles of Galactosamine-hyaluronic acid-Vitamin E succinate (Gal-HA-VES) for targeting delivery of NCTD to HCC. NCTD/Gal-HA-VES micelles could quickly release NCTD in acidic (pH 5.5) and rich-hyaluronidase tumors tissue, thereby showing higher cytotoxicity toward CD44-overexpressing MCF-7 cells, MCF-7/Adr cells and ASGPR overexpressing HepG2 cells by CD44 receptor and ASGPR mediated endocytosis. Moreover, Gal-HA-VES could act as a P-glycoprotein (P-gp) inhibitor to block drug efflux in MCF-7/Adr cells. In vivo study also demonstrated that this micelle improved tumor targeting ability and antitumor effect with low toxicity.

#### ITGA5 receptor-based NCTD delivery

**Fig. 5:**
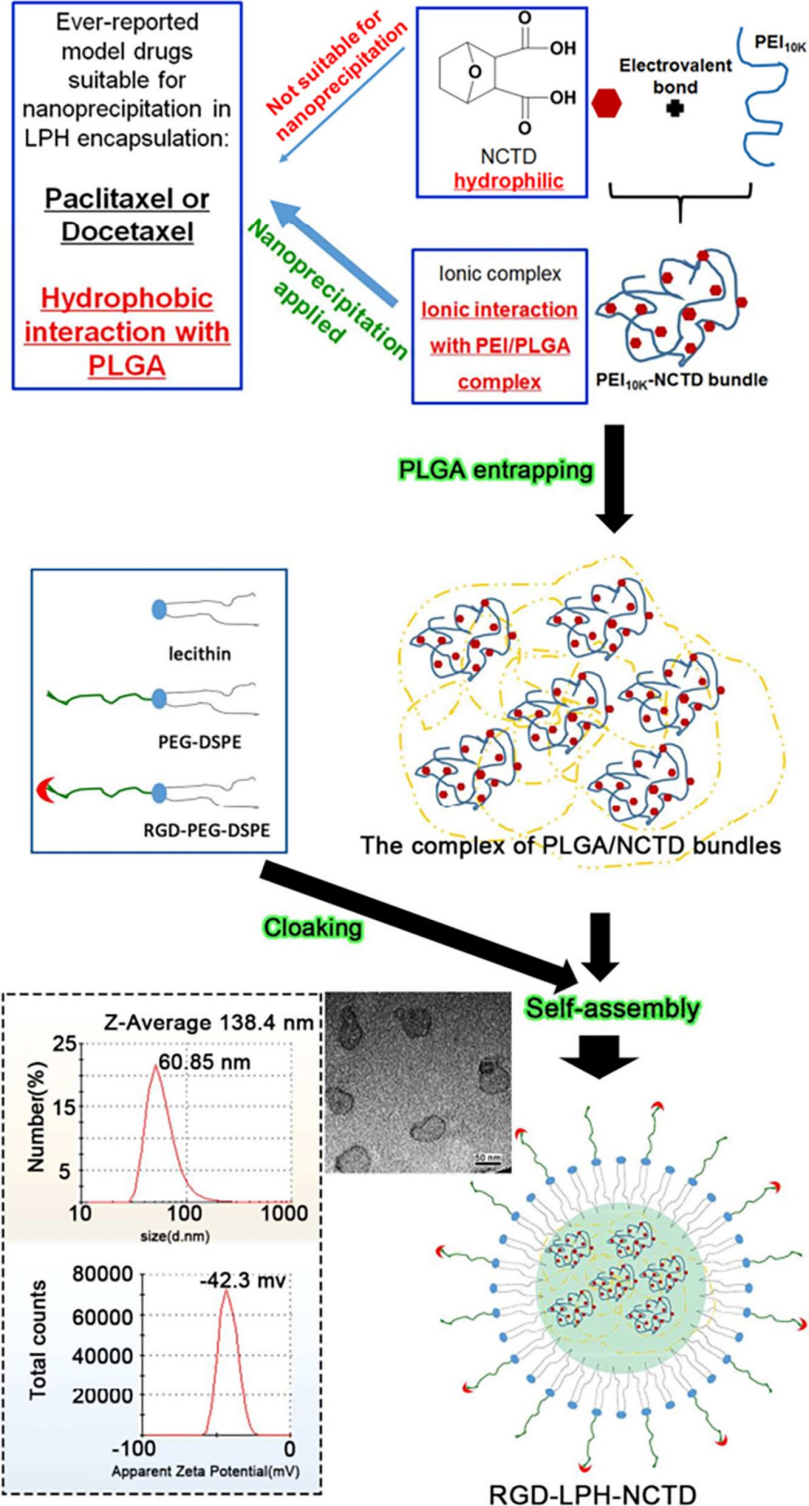
RGD-anchoring lipid-polymer hybrid (LPH) nanoparticle encapsulating NCTD (RGD-LPH-NCTD) schematic illustration and characterization. Reproduced with permission from reference [135]. Copyright 2019, Pergamon

Cancer stem cells (CSCs) play key roles in cancer metastasis [130]. The canonical Wnt/β-catenin pathway plays critical roles in CSCs generation and maintenance [131, 132]. Therefore, strategies targeting CSCs by specifically inhibiting the Wnt/β-catenin pathway may greatly reduce cancer metastasis. Several studies have suggested that NCTD may inhibit the β-catenin pathway through its potent inhibition of protein phosphatases, thereby impairing the stemness of pancreatic and other cancer cells [133, 134]. Moreover, based on the high expression of ITGA5 in triple-negative breast cancer (TNBC) and its lung metastases, ITGA5 ligands such as RGD motif (Arg-Gly-Asp) modified nano delivery system can actively deliver drugs to TNBC. Based on this, Li et al. [135] reported an ITGA5-targeting diacidic norcantharidin-loaded lipid-polymer hybrid (LPH) nanoparticle (RGD-LPH-NCTD) for targeted therapy of TNBC (Fig. 5). It is worth noting that RGD-modified LPH showed more accumulation than LPH in orthotopic TNBC tumor and their lung metastases. Compared with NCTD and LPH-NCTD, RGD-LPH-NCTD more significantly reduced orthotopic TNBC tumor growth and metastasis by attenuating β-catenin. Therefore, RGD-LPH-NCTD may offer a promising approach for the treatment of metastatic TNBC by specially down-regulating β-catenin.

#### FA receptor-based NCTD delivery

The FA receptor is a single-chain membrane glycoprotein receptor that binds folate with high affinity and transports it into cells. Studies have found that FA receptors are underexpressed in normal tissues, but overexpressed in various tumor cells (such as liver cancer, colon cancer, lung cancer, prostate cancer, ovarian cancer, and breast cancer), so they can be used as targets for tumor therapy [136]. Liu et al. [137] prepared a FA-conjugated NCTD-loaded stealth niosomes with average size of 100.87 nm and average EE of 52.3%. The release t_1/2_ of FA niosomes at pH 7.4 was 1.98 times higher than that at pH 5.0, indicating that FA vesicles have pH-sensitive properties and are more likely to release drugs in the tumor microenvironment. They also increased the tumor cell uptake towards the drug and enhanced tumor cell cytotoxicity (IC_50_ = 46 µg/mL) than non-targeted stealth niosomes (IC_50_ = 148 µg/mL). Liu et al. [138] also developed a FA-modified DM-NCTD-loaded PEGylated (DM-NCTD/FA-PEG) liposome for targeting HCC. Compared with DM-NCTD/PEG liposomes [relative intake rate (Re) 4.86, tissue/tumor-targeting efficacy (Te) 12.81%, relative targeting efficiency (R_Te_) 2.36, and peak concentration ratio (Ce) 4.78], DM-NCTD/FA-PEG liposomes showed enhanced tumor-targeting efficiency (Re 9.25, Te 24.44%, R_Te_ 4.50, and Ce 9.24). Moreover, the antitumor activity of DM-NCTD/FA-PEG liposomes on H22 tumor-bearing mice was enhanced, and tumor-cell apoptosis was more pronounced and there was no obvious toxicity to the tissues of model mice or to the liver tissue of normal mice.

## Physicochemical targeted drug delivery systems

Nanotechnology-based targeted drug delivery has shown promising results in preclinical animal models. However, drug delivery systems that rely on the EPR effect and ligand recognition still have problems in clinical application, which may be limited by various tumor microenvironmental factors such as tumor heterogeneity, hypoxia, endosomal escape and the facile inactivation of many targeting ligands and the difficulty of nanocarriers in controlling drug release [139, 140]. Therefore, the drug delivery system is designed according to the tumor microenvironment to simulate biological responsiveness and realize the on-demand response release of drugs, which is beneficial to the precise treatment of tumors. Such delivery systems can release drugs in response to specific physical or chemical conditions, so they are also called physicochemical targeted drug delivery systems [141, 142]. Existing physicochemical targeted delivery systems for NCTD mainly rely on chemical endogenous stimuli (pH) of tumor microenvironment and exogenous physical stimuli (temperature, charge, light and interventional embolization) to achieve targeted drug delivery (Table 4).

**Table 4:** Physicochemically targeted drug delivery system for norcantharidin

Drug delivery system	Composition	Stimulus-response properties	Efficacy	Ref., year
NCTD-loaded thermosensitive in-situ gel	28% poloxamer 407, 1.6% poloxamer 188, 0.1% hydroxypropyl methyl cellulose (HPMC), NCTD	Thermosensitive: gelation temperature: 34 ℃, dissolution time: 210 min; sustained-release property in vitro	-	[145], 2017
NCTD-loaded thermosensitive in-situ gel	62.5 mg NCTD, 7 g poloxamer 407, 0.4 g poloxamer 188, 0.025 g HPMC	Thermosensitive: gelation temperature and time: (33.9–34.1) ℃, (101–103) s; viscosity: > 100,000 mPa·s (37 ℃); sustained-release property in vitro	H22 tumor inhibition rate: NCTD (*ip.*, once daily, 2 mg/kg) < NCTD-loaded thermosensitive in-situ gel (intratumoral injection, once every 3 days, 3.3 mg/kg) < NCTD-loaded thermosensitive in-situ gel (intratumoral injection, once every 3 days, 6.6 mg/kg) < NCTD-loaded thermosensitive in-situ gel (intratumoral injection, once every 3 days, 9.9 mg/kg)	[146], 2017
NCTD-loaded thermosensitive in-situ gel	62.5 mg NCTD, 7 g poloxamer 407, 0.4 g poloxamer 188, 0.025 g HPMC	Thermosensitive: gelation temperature: 34 ℃, dissolution time: 3.5 h	H22 tumor inhibition rate: NCTD (*ip.*, once daily, 2 mg/kg) < NCTD-loaded thermosensitive in-situ gel (intratumoral injection, once every 3 days, 3.3 mg/kg) < NCTD-loaded thermosensitive in-situ gel (intratumoral injection, once every 3 days, 6.6 mg/kg) < NCTD-loaded thermosensitive in-situ gel (intratumoral injection, once every 3 days, 9.9 mg/kg); survival time ↑; inhibit VEGF and CD44 expression ↑	[147], 2019
NCTD-loaded metal-organic framework (IRMOF-3) coated with a poloxamer thermosensitive gel (NCTD-IRMOF-3-Gel)	15 mg NCTD-IRMOF-3 (NCTD, IRMOF-3), 3 mL freeze-dried protective agent (4% mannitol and 2% poloxamer)	Thermosensitive; NCTD release (5 h): sustained-release effect: NCTD (90% NCTD) > NCTD-IRMOF-3 (50% NCTD) > NCTD-IRMOF-3-Gel (30% NCTD)	Hepa1-6 cell: cell inhibition rate: NCTD-IRMOF-3-Gel > NCTD, NCTD-IRMOF-3; block cell cycle in the S and G2/M phases; cell apoptosis (48 h, 50 µg/mL): NCTD-IRMOF-3-Gel > NCTD-IRMOF-3 > NCTD	[148], 2021
Thermosensitive hydrogel co-loaded with NCTD nanoparticles and doxorubicin (NCTD-NPs/Dox Gel)	85 mg NCTD-NPs (PCEC copolymers, NCTD), 2 mg Pluronic F127 (PF127), 30 mg Dox	Thermosensitive: gelation temperature: 29.4 ℃; NCTD release: sustained-release effect: NCTD (48 h, 94.2% NCTD) > NCTD-NPs (168 h, 84.5% NCTD) > NCTD-NPs/Dox Gel (168 h, 47.8% NCTD)	HepG2 cells: IC_50_: NCTD-NPs/Dox Gel < free NCTD/Dox; H22 tumor inhibition rate (intratumoral injection, 0.1 mL, once a week for two times): free NCTD/Dox (2.5 mg/kg free NCTD and 10 mg/kg free Dox) < NCTD-NPs/Dox Gel (equivalent to 2.5 mg/kg free NCTD and 10 mg/kg free Dox); median survival time: free NCTD/Dox group (53 days) < NCTD-NPs/Dox Gel (67 days); no visible tissue damage, inflammation, or lesions; inhibit tumor proliferation and angiogenesis: Ki-67-positive cells: NCTD-NPs/Dox Gel (22.46 ± 2.51) % < free NCTD/Dox (38.77 ± 4.85) %, CD31-positive microvessel density (MVD): NCTD-NPs/Dox Gel (7.67 ± 0.94) < free NCTD/Dox (15.34 ± 2.05)	[149], 2021
Lactosyl-NCTD (Lac-NCTD) phospholipid complex (LPC) loaded liposome (pH-LPC-lips)	40 mg soybean phosphatidylcholine, 10 mg cholesterol, 4 mg Lac-NCTD LPC, 2 mL 0.02% CMCT solution	pH sensitivity: cumulative release of NCTD: pH = 5.2 > pH = 6.8 > pH = 7.4	-	[152], 2011
pH-LPC-lips	40 mg soybean phosphatidylcholine, 10 mg cholesterol, 4 mg Lac-NCTD LPC, 2 mL 0.02% CMCT solution	pH sensitivity	HepG2 cells: IC_50_: pH-LPC-lips (0.094 µmol/mL) < Lac-lips (0.140 µmol/mL) < Lac-NCTD (0.351 µmol/mL); cellular uptake: pH-LPC-lips > Lac-lips > Lac-NCTD; H22 tumor inhibition rate (*ip.*, once daily, 10 days): NCTD (2.0 mg/kg) = 31.2% < Lac-NCTD (6.6 mg/kg) = 38.3% < Lac-lips (6.6, 13.2 mg/kg) = 56.6%, 76.9% < pH-LPC-lips (6.6, 13.2 mg/kg) = 63.6%, 85.3%; the accumulation of NCTD in liver and tumor tissues ↑	[153], 2014
Cross-linked polymer nano-cooperative prodrugs (PPD-NPs)	PPD [cisplatin/NCTD dual drug small molecule (Pt (IV)-1), EDC, NHS, DMF, polyorthoester main chain (POEAd-NH_2_)], DMSO, phosphate buffer (pH = 7.4, 50 mM)	pH sensitivity: Pt release (24 h): pH = 5.0 + 10 mM GSH (80%) > pH = 6.8 (30%) > pH = 7.4 (15%); H22 and HepG2 cellular uptake: pH = 6.8 > pH = 7.4	H22 and HepG2 cells: IC_50_: PPD-NPs (pH = 6.8) < PPD-NPs (pH = 7.4) < cisplatin + NCTD < Pt (IV)-1; induce cell apoptosis; concentration of Pt in mouse blood and tumors: PPD-NPs > Pt (IV)-1 > cisplatin + NCTD; H22 tumor inhibition rate (*iv.*, 6 mg/kg): PPD-NPs > Pt (IV)-1 > cisplatin + NCTD; Pt-DNA ↑, PPA2 activity ↓; inhibit HepG2 tumor growth (PPD-NPs); heart and kidney toxicity ↓	[156], 2021
NCTD-loaded TPP-PEG-PCL nanomicelles (NCTD@TPP- PEG-PCL)	10 mg NCTD, 100 mg TPP-PEG-PCL, PBS	Attraction of positive and negative charges: TPP cations (positive charge), cell membranes and mitochondrial membranes (negative charge)	NCTD@TPP-PEG-PCL/NCTD@PEG-PCL/NCTD: t_1/2_, AUC, MRT ↑; SMMC-7721 cells: cellular uptake ↑, mitochondrial targeting ability ↑, lysosome escape ↑; inhibit cell growth and induce cell apoptosis: NCTD@TPP-PEG-PCL > NCTD@PEG-PCL > NCTD; mitochondrial membrane potential ↓, ROS ↑, Bcl-2 ↓, Bax ↑	[159], 2020
Charge- reversal polymer nano-modulator (SP_DMC_N)	The amphiphilic semiconducting polymer with acid-labile DMC-incorporating conjugates on the surface (SP_DMC_)	pH sensitivity and charge conversion: zeta potential: -17 mV (pH 7.4), + 7 mV (pH 6.5), + 12 mV (pH 5.0); ^1^O_2_ generation: pH 6.7 > pH 7.4; tumor penetration ↑	CT26 cells: SP_DMC_N (IC_50_ = 22 µg/mL) < SP_SA_N (IC_50_ = 30.8 µg/mL); PP2A ↓, PI3K/AKT ↑, mTORC1 ↑, FOXP3 ↓, Treg ↓; CT26 primary and distant tumor inhibition rate (*iv.*, 2 mg [SPN]/kg, laser condition: 808 nm, 0.3 W/cm^2^, 8 min): SP_DMC_N > SP_SA_N; caspase-3 ↑; favorable in vivo biosafety and biocompatibility; CD8^+^/CD4^+^ ratio ↑; granzyme B ↑; HMGB1 (ICD) ↑; CD11c^+^CD80^+^CD86^+^ DCs (DC maturation) ↑; IFN-γ, IL-6, TNF-α ↑; CD4^+^CD25^+^FOXP3^+^ Tregs cells among CD3^+^ T cells ↓; CD8^+^/Treg ratio ↑; CTL ↑	[160], 2021
Light-activatable dual prodrug polymer nanoparticle (DPP NP)	DP monomer (100 mg, 1.0 eq), EDC (58.8 mg, 2.3 eq), NHS (35.5 mg, 2.3 eq), DMF (8 mL), EDA (7.9 mg, 0.98 eq), mPEG_2 K_-NH_2_ (20 mg, 0.08 eq)	Light responsiveness: drug release could be precisely controlled by visible light (upon periodic irradiation and upon preirradiation)	HeLa cells: cellular uptake ↑, p-AKT protein and Pt-DNA adducts (light irradiation) ↑, IC_50_ (light irradiation): DPP NP (53.5 µM) < DP (64.1 µM) < NCTD (108 µM) < Pt(IV) (170 µM); KM mice bearing U14 tumor: Pt DMCT imaging of DPP NP; U14 tumor inhibition rate (*iv.*, 3 mg Pt/kg on days 0, 3 and 6, light irradiation: 430 nm, 20 mW/cm^2^, 30 min): 75% of mice were fully cured, apoptosis ↑, survival rate ↑	[162], 2019
NCTD-loaded PLGA-alginate microsphere (NPAM)	2 mL ethyl acetate containing 0.5 g PLGA and 0.3 g NCTD, 50 g aqueous solution containing sodium alginate, 75 g isooctane containing 2.54 g Span 85, 5 g aqueous solution containing 1.36 g Tween 85, 20 g 15% (w/w) calcium chloride solution	Chemoembolization; average size: (46.9 ± 5.4) µm (PLGA: alginate = 1: 3, w: w); drug released by burst effect ↓; disintegration time: about 4 days	SMMC-7721 cells: NPAM: IC_50_ (24 h, 48 h, 72 h) = 110.2, 70.6, 35.5 µg/mL; the growth rate of the tumor after treatment: 1.5 mL/kg 0.03% (w/v) NCTD solution (12.4) > 10 mg/kg blank PLGA-alginate microspheres (BPAM) (10.1) > 10 mg/kg NPAM (1.1); survival time and survival rate: 1.5 mL/kg 0.03% (w/v) NCTD solution (15.8 ± 2.0 days, 15.8%) < 10 mg/kg BPAM (16.5 ± 3.0 days, 20.7%) < 10 mg/kg NPAM (31.0 ± 3.9 days, 126.8%)	[165], 2006
NCTD-chitosan microsphere (NCTD-CS-MS)	NCTD, 5% dilute acetic acid, CS, liquid paraffin containing Span-80, 25% glutaraldehyde-saturated toluene solution	Chemoembolization; average size: (143.54 ± 4.24) µm, within 60–200 μm account for 87%; sustained-release property (cumulative release rate in 7 days reaches about 60%)	Establishment of a rabbit VX-2 liver cancer model by ultrasound-guided puncture (NCTD: 1.0 mg/kg, NCTD-CS-MS: 10.09 mg/kg): survival time and life prolonging rate: NCTD (16.23 ± 0.45 days, 20.69 ± 3.35%) < NCTD-CS-MS (25.73 ± 0.60 days, 91.33 ± 4.48%); tumor growth rate: NCTD-CS-MS (9.72%) < NCTD (12.9%)	[166], 2010
NCTD sustained-release microsphere (NCTD-MS)	NCTD, 10 mL 2.0% sodium alginate, 0.15 g nanoscale calcium carbonate, 50 mL liquid paraffin containing 1% Span-80, 1.0 mL glacial acetic acid, 2% CS solution; drug/carrier (sodium alginate + calcium carbonate) = 0.8:1 (w/w)	Chemoembolization; average size: (309.75 ± 2.19) µm; sustained-release property; NCTD release: NCTD-MS (normal saline, pH 7.4 PBS) = 54%, 32% (3 h) and 80% (24 h) < NCTD (normal saline, pH 7.4 PBS) = 100% (3 h)	-	[167], 2011
Lipidic solid dispersion of NCTD-loaded alginate/CS microsphere (LSD/NTCD-ACM)	NCTD, sodium alginate, 50 mL liquid paraffin containing Span-80, CS, calcium chloride, glutaraldehyde, insect wax	Chemoembolization; skeleton type sustained release effect; NCTD release: LSD/NTCD-ACM (120–200 μm) = (53.05 ± 2.73) % (5 days) < LSD/NTCD-ACM (60–120 μm) = (73.65 ± 0.94) % (5 days) < LSD/NTCD = (82.44 ± 1.36) % (5 days) < NCTD-ACM (120–200 μm) = 80% (24 h) < NCTD = 100% (3 h)	Establishment of a rabbit VX-2 liver cancer model by ultrasound-guided biopsy needle method: survival time and life prolonging rate: NCTD [(26.67 ± 0.58) days, -(0.03 ± 4.55) %] < LSD/NTCD-ACM (60–120 μm) [(39.49 ± 0.51) days, (43.81 ± 4.34) %] < LSD/NTCD-ACM (120–200 μm) [(43.37 ± 0.45) days, (57.94 ± 5.76) %]; tumor growth rate: LSD/NTCD-ACM (60–120 μm) = (7.76 ± 0.41) % < LSD/NTCD-ACM (120–200 μm) = (9.56 ± 0.37) % < NCTD = (13.37 ± 1.63) %	[168, 169], 2011, 2013
NCTD-loaded silk fibroin/CS microsphere (NCTD-SF/CS-MS)	NCTD, silk fibroin, CS, dilute acetic acid, liquid paraffin containing Span-80, glutaraldehyde	Chemoembolization; average size: (184 ± 5) µm; sustained-release property; NCTD release: NCTD-SF/CS-MS < NCTD-CS-MS	Establishment of a rabbit VX2 liver tumor model by ultrasound-guided percutaneous puncture: survival time and life prolonging rate: NCTD [(26.67 ± 0.58) days, -0.03%] < blank-microsphere (B-MS) (100–200 μm) [(35.79 ± 0.26) days, 25.83%) < NCTD-CS-MS (100–200 μm) [(37.51 ± 0.46) days, 31.89%] < NCTD-SF/CS-MS (100–200 μm) [(40.29 ± 0.34) days, 41.66%]; tumor growth rate: NCTD-SF/CS-MS (100–200 μm) < NCTD-CS-MS (100–200 μm) < B-MS (100–200 μm) < NCTD; tumor necrosis rate ↑	[172], 2013
NCTD-N-chitosan/silk fibroin microsphere (NCTD-N-CS/SF-MS)	NCTD, silk fibroin, N-CS, dilute acetic acid, liquid paraffin containing Span-80, glutaraldehyde	Chemoembolization; average size: (117 ± 4.3) µm; sustained-release property; NCTD release: NCTD-N-CS/SF-MS = 30% (40 min), 60% (7 days) < NCTD = 100% (40 min)	Establishment of a rabbit VX2 liver tumor model by ultrasound-guided percutaneous puncture: survival time and life prolonging rate: NCTD (23.25 days, -1.25%) < NCTD + N-CS/SF-MS (31 days, 31.91%) < N-CS/SF-MS (34 days, 44.68%) < NCTD-N-CS/SF-MS (36.25 days, 54.25%); tumor inhibition rate and tumor cell necrosis rate: NCTD-N-CS/SF-MS [85.01%, (56.78 ± 0.84) %] > NCTD + N-CS/SF-MS [62.98%, (52.23 ± 0.64) %) > N-CS/SF-MS [58.16%, (49.63 ± 1.02) %] > NCTD [27.22%, (19.35 ± 0.92) %];	[173, 174] 2013, 2014

### Thermosensitive hydrogel-based NCTD delivery

Thermosensitive hydrogels, which are free-flowing liquids at room temperature while convert to semi-solid gels at body temperature, have been widely used for controlled drug delivery due to their sustained-release properties [143]. Poloxamer (Pluronic), a poly(ethylene oxide)-poly(propylene oxide)-poly(ethylene oxide) (PEO-PPO-PEO) triblock copolymer, exhibits amphiphilic properties and undergoes a thermoreversible sol-gel transition that is used widely in the thermo-gelling system [144]. Recently, many studies have showed that delivery of NCTD through poloxamer-based gel systems can increase drug concentration at administration sites, reduce adverse reactions and irritation, thereby improving efficacy and safety.

Zhou and Xie et al. [145–147] prepared a NCTD-loaded thermosensitive in situ gel using poloxamer 407, poloxamer 188 and hydroxypropyl methyl cellulose (HPMC) as drug carriers. This gel solution could rapidly undergo a phase transition to form a semi-solid gel at 34 °C, resulting in stable and slow drug release. Compared with NCTD injection, treatment with NCTD thermosensitive gel showed enhanced antitumor activity and better improved survival of H22 tumor-bearing mice by inhibiting VEGF and CD44 expression. Li et al. [148] established a NCTD-loaded multifunctional metal-organic framework (IRMOF-3) coated with a poloxamer thermosensitive gel (NCTD-IRMOF-3-Gel), which prolonged the action time of the drug from the IRMOF carrier, therefore increased the antitumor effect of NCTD by blocking the Hepa1-6 cell cycle in the S and G2/M phases and inducing cell apoptosis.

**Fig. 6:**
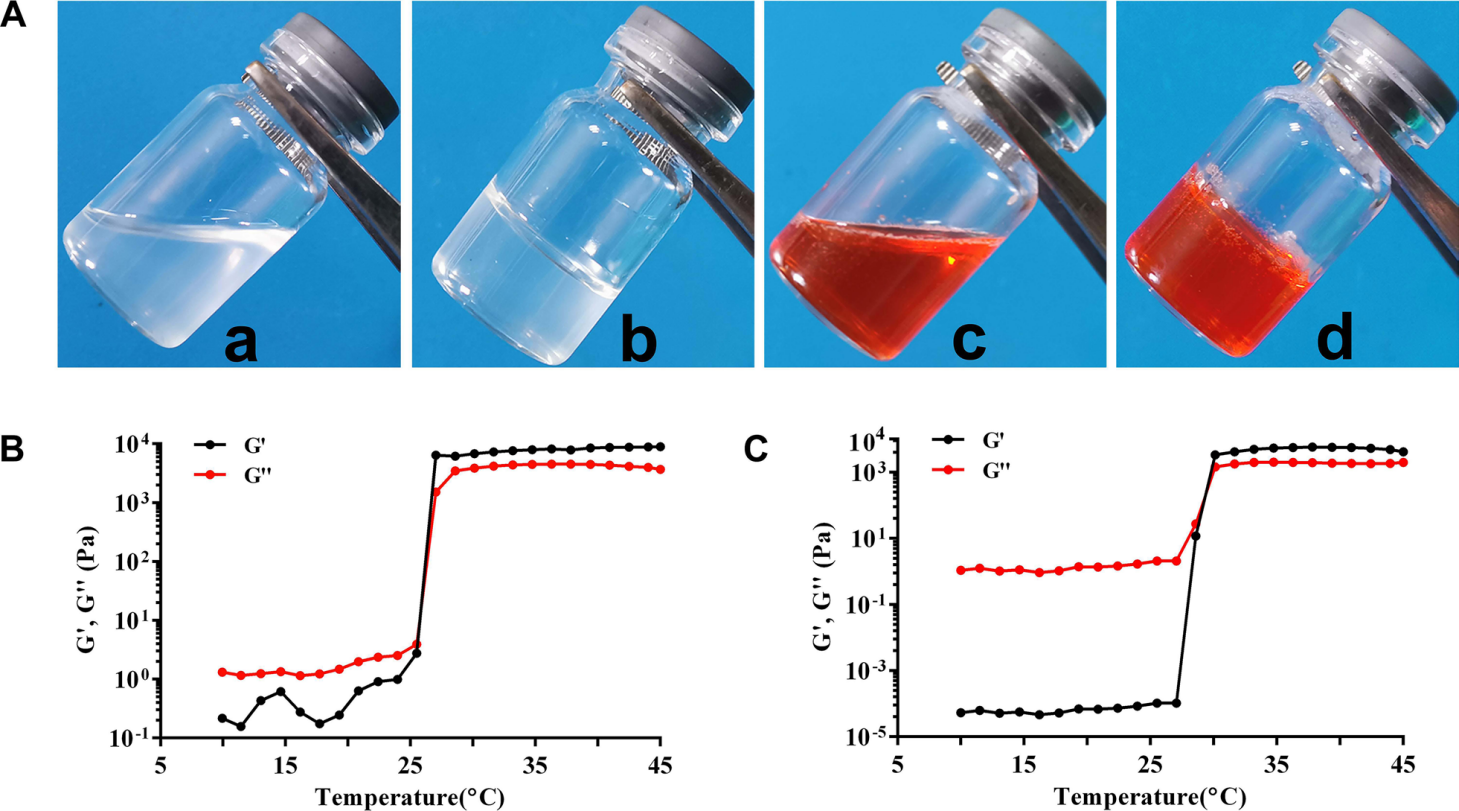
Thermal-sensitive analysis of the PF127 hydrogel drugs. **A** Morphology of the blank hydrogel (a) and drug-loaded hydrogel (c) at room temperature; the blank hydrogel (b) and drug-loaded hydrogel (d) at 37 °C. Rheological analysis as a function of temperature: **B** the blank hydrogel and **C** drug-loaded hydrogel. *PF127* Pluronic F127. Reproduced with permission from reference [149]. Copyright 2021, DOVE Medical Press

Gao et al. [149] developed a Pluronic F127 (PF127)-based thermosensitive hydrogel (NCTD-NPs/Dox Gel) by encapsulating NCTD-loaded poly (ε-caprolactone)-PEG-poly (ε-caprolactone) (PCL-PEG-PCL, PCEC) nanoparticles (NCTD-NPs) with Dox. This hydrogel showed good thermal sensitivity, sustained drug release effect, and enhanced cytotoxicity in HepG2 cells. Thermal-sensitive analysis of the PF127 hydrogel was showed in Fig. 6. Moreover, intratumoral administration of the NCTD-NPs/Dox Gel significantly inhibited tumor proliferation and angiogenesis by reducing the expression of Ki-67 and CD31, relieved side effects, and extended survival of H22 tumor-bearing mice.

### pH-sensitive-based NCTD delivery

The pH of normal human tissue is around 7.4, while cancer cells have a high glycolysis rate under aerobic or anaerobic conditions. Glycolysis converts glucose into lactic acid, resulting in an acidic tumor microenvironment (pH 6.0-7.2), and a lower pH of endosomes and lysosomes in tumor cells, ranging from 4.0 to 6.0. Using the above pH changes to design nano drug delivery systems with pH-responsive drug release function is helpful to achieve targeted drug release in tumor tissue [150].

CMCS has both cationic groups (-NH_3_^+^) and anionic groups (-COO^−^), and is an amphiphilic polyelectrolyte. It is negatively charged in the physiological pH and positively charged in the acidic environment of the tumor. CMCS undergoes conformational changes at different pH values, leading to the destabilization of the liposome bilayer and the release of the drug [151]. A research group synthesized a Lac-NCTD phospholipid complex (LPC) loaded liposome (pH-LPC-lips), in which soybean phosphatidylcholine was employed to increase the liposolubility of Lac-NCTD and CMCS was incorporated onto the liposomal surface by electrostatic adsorption. The results of the in vitro and in vivo studies proved the improvement of therapeutic efficacy and tumor targeting of pH-LPC-lips on HCC [152, 153].

Polyorthoesters are acid-sensitive biomaterials that can control the release of drugs in response to the slightly acidic environment inside and outside the tumor cells [154]. In addition, cisplatin and NCTD can achieve synergistic antitumor effect when combined with a molar ratio of 1:2 [155]. Based on this, Wang et al. [156] synthesized the small cisplatin-NCTD prodrug molecule in the optimal ratio, then cross-linked the main chain polyorthoester to obtain the amphiphilic cross-linked polymer prodrug, and further self-assembly formed a nano-prodrug coordinated delivery system. The prepared nano-prodrug could release about 15% of the drug at pH = 7.4 within 24 h, release about 30% of the drug at pH = 6.8, and release about 80% of the drug at pH = 5.0/GSH, indicating that it has excellent pH responsive drug release properties. On H22 and HepG2 liver cancer models, the nano-prodrug can not only exist stably in the blood circulation, but also efficiently respond to the intracellular and extracellular physiological microenvironment of the tumor to enhance the ability of tumor-targeted enrichment, cellular uptake, and synergistic killing of cancer cells, while reducing toxic side effects on major organs.

### Charge-based NCTD delivery

(3-carboxypropyl)triphenylphosphonium bromide (TPP) cations are positively charged, and cell membranes and mitochondrial membranes are negatively charged. In view of the attraction of positive and negative charges, TPP can be used to mediate tumor drugs to overcome the barriers of cell membranes and mitochondrial membranes, and ultimately target into the mitochondria [157, 158]. Han et al. [159] prepared a TPP-modified NCTD-loaded PEG-PCL nano-micelle by thin-film hydration method, with a particle size of (16.8 ± 0.2) nm and a zeta potential of (14.3 ± 0.2) mV. NCTD-loaded TPP-PEG-PCL nano-micelle can promote the cellular uptake of NCTD, escape lysosomal capture, and finally target aggregation at the mitochondrial site. This nano-micelle also had a good effect on promoting apoptosis of liver tumor cells by reducing mitochondrial membrane potential, increasing intracellular reactive oxygen species (ROS) levels, increasing pro-apoptotic protein Bcl-2, and reducing resistance, which is a potentially effective drug delivery system for targeting tumor cell mitochondria.

**Fig. 7:**
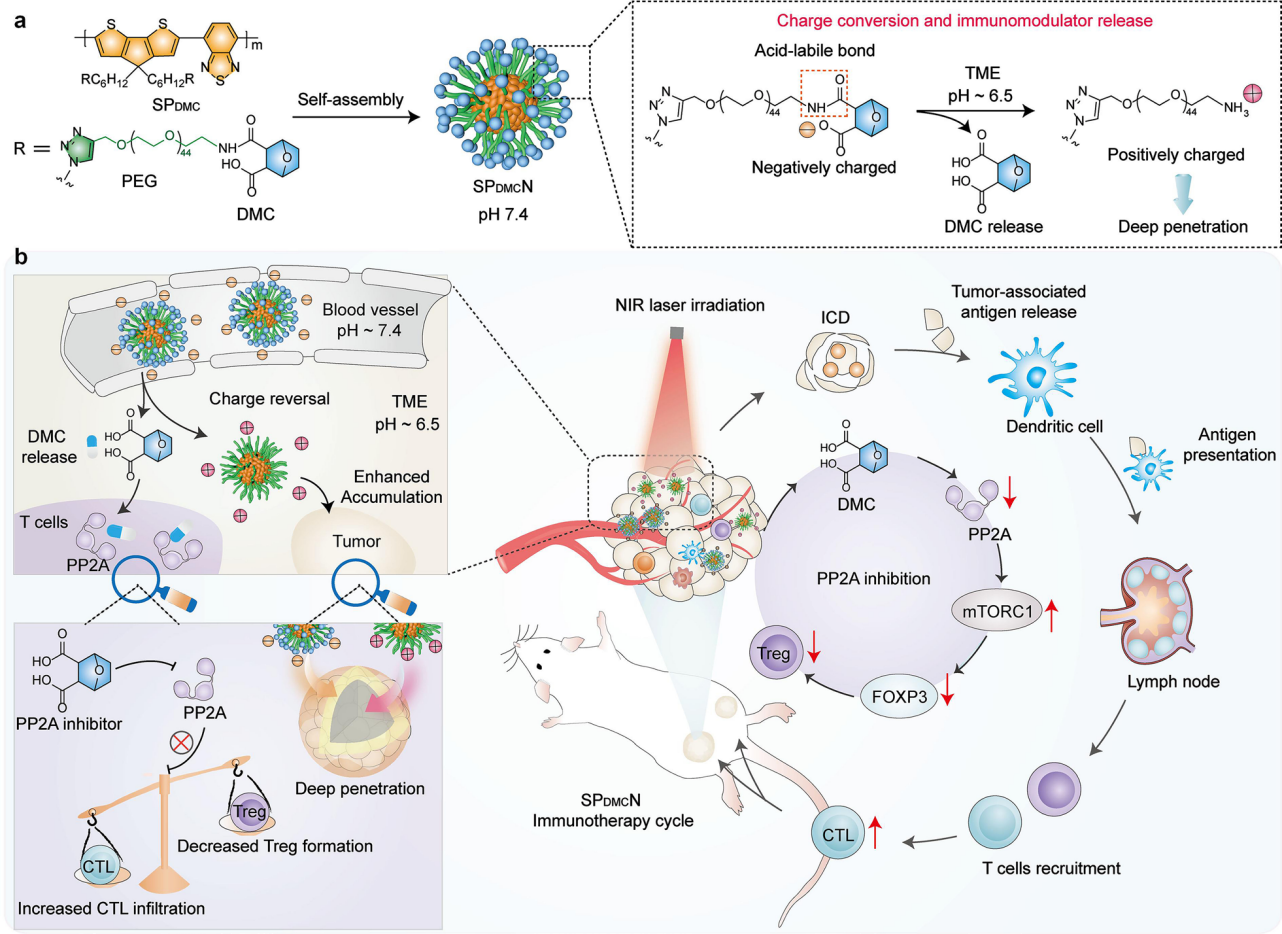
Schematic illustration of charge-reversal semiconducting polymer nano-modulator (SP_DMC_N)-mediated photodynamic cancer immunotherapy. **a** Chemical structure and self-assembly of SP_DMC_N, and the mechanism of pH-responsive charge-reversal and immunomodulator (NCTD) release. **b** Illustration of SP_DMC_N-mediated synergistic immunotherapy, including acidic tumor microenvironment induced (NCTD) release, photoirradiation of SP_DMC_N for ICD induction, and immune cell modulation by NCTD. Reproduced with permission from reference [160]. Copyright 2021, JOHN/WILEY & SONS, INC

Moreover, He et al. [160] designed an acidic tumor microenvironment-responsive charge-reversal polymer nanoparticle (SP_DMC_N) that can specifically release immunomodulator (NCTD) and enhance tumor penetration for combinational photodynamic cancer immunotherapy. SP_DMC_N is constructed by conjugating NCTD to the side chains of a semiconducting polymer via an acid-liable β-carboxylic amide bond. SP_DMC_N had a size of about 12 nm at physiological pH (zeta potential: -17 mV), while in the acidic condition, the acid-labile amides of SP_DMC_N hydrolyzed into free amines to form SP-NH_2_ (zeta potential: +12 mV), which resulted in deep tumor penetration of the nanoparticles and localized release of NCTD. Upon near-infrared laser irradiation, the SP core of SP_DMC_N generated ^1^O_2_ to ablate the primary tumors, simultaneously inducing immunogenic cell death (ICD) and promoting dendritic cells (DCs) maturation. In addition, NCTD specifically inhibited protein phosphatase 2 (PP2A), which significantly decreased regulatory T lymphocytes (Tregs), and in turn remarkably promoted cytotoxic T lymphocytes (CTLs) infiltration, affording a significant increase in CD8^+^/Treg ratio. Therefore, SP_DMC_N showed superior antitumor efficacy against both primary and distant tumors with a tumor inhibition rate over 88% and low adverse reactions (Fig. 7).

### Light-based NCTD delivery

**Fig. 8:**
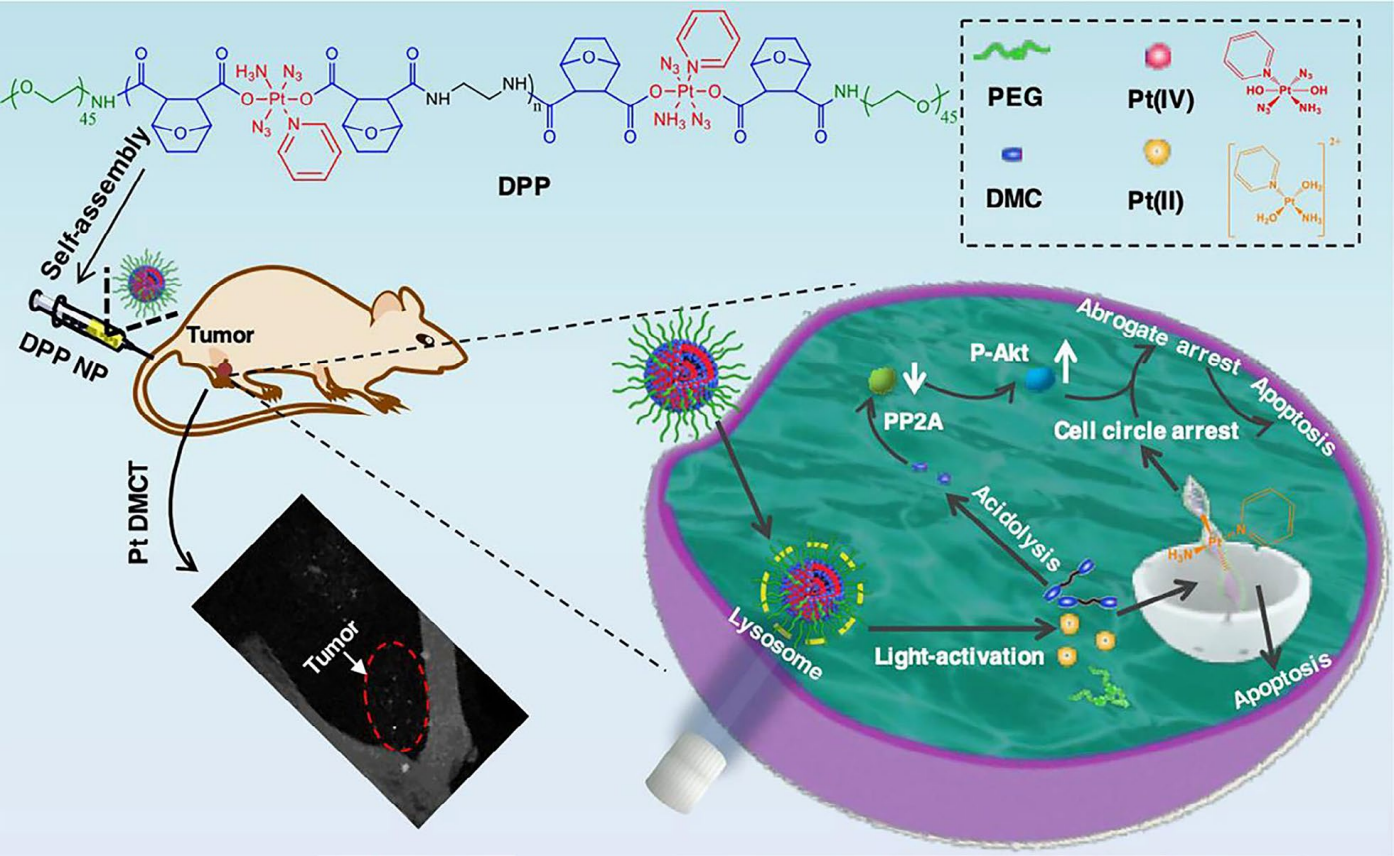
Schematic diagram of the light-activatable dual prodrug polymer nanoparticle (DPP NP) for precise synergistic chemotherapy guided by Pt drug-mediated computed tomography (DMCT) imaging. Reproduced with permission from reference [162]. Copyright 2019, Elsevier

Light-responsive polymeric delivery systems are attractive drug delivery systems due to their inactive and stable under normal conditions but can release intact drugs in response to light, thus providing better control on drug release and resulting in less side effects [161]. Wang et al. [162] designed a light-responsive dual prodrug polymer nanoparticle (DPP NP) for precise synergistic chemotherapy guided by drug-mediated computed tomography (DMCT) imaging, in which NCTD was conjugated to a light-activatable Pt(IV) prodrug to construct the dual prodrug (DP) monomer. After endocytosis and visible light irradiation, the polymer backbone is cleaved and the Pt(IV) prodrug is activated to release the Pt(II) drug, resulting in DNA damage. Afterwards, NCTD released in the acid endo/lysosome microenvironment would block the repair of damaged DNA by inhibiting PP2A, thereby exhibiting synergistic chemotherapy (Fig. 8). Notably, the ratio of the Pt(II) drug and NCTD in DPP NP was fixed at an optimal value (Pt/NCTD = 1/2) even after endocytosis. Moreover, the DPP NP could be used as a CT imaging contrast agent to monitor the distribution of drugs due to the high Pt content, thereby guiding the intensity and time of light exposure. Guided by Pt DMCT imaging, this nanoparticle exhibited excellent antitumor activity with complete cure in 75% of tumors.

### Chemoembolization-based NCTD delivery

Clinically, transcatheter arterial embolization (TAE) can be used as an effective method of non-surgical treatment for patients with unresectable advanced cancer or patients who are unwilling to undergo surgery. TAE could effectively inhibit tumor growth by injecting embolic materials into the arteries of tumors, cutting off the blood supply to the tumor, making it unable to obtain the oxygen and nutrients needed for survival. In TAE technology, the choice of embolization material determines the therapeutic effect of interventional embolization [163]. Drug embolization microspheres not only have a large specific surface area, but also can combine chemotherapy and embolization, which has become a hot spot of current research [164]. In recent years, a variety of NCTD-loaded embolic microspheres have been developed, and have shown good therapeutic effect in the treatment of liver cancer.

Liu et al. [165] prepared a NCTD-loaded PLGA-alginate microsphere (NPAM) for synergistic chemotherapy and embolization. NPAM were more effective than NCTD solution and blank microspheres in inhibiting tumor growth and extending survival in a liver cancer tumor model. Song et al. [166] prepared a NCTD-CS microsphere (NCTD-CS-MS) by emulsification-chemical cross-linking method. The average particle size of the microspheres was (143.54 ± 4.24) µm. The microspheres were used as embolizing agent for hepatic artery embolization therapy for rabbit VX-2 hepatocarcinoma. Since the microspheres can be embolized to the presinusoidal arterioles through the hepatic artery administration, the nutrition source of the tumor can be well cut off. While exerting the embolization effect, the drug in the microspheres can be continuously and slowly released locally in the liver cancer, so that the local concentration can be maintained at a high level, which has a good anticancer effect and can significantly reduce the systemic toxicity. Zhou et al. [167] prepare a NCTD sustained-release microsphere (NCTD-MS) for hepatic arterial embolism by inner gel technique with alginate-chitosan as carrier. The microsphere was of average diameter (309.75 ± 2.19) µm. Drug release rate of the microsphere in phosphate buffer solution and normal saline was 80% in 24 h, while NCTD raw material released completely in 3 h. Zhou and Zhang et al. [168, 169] also prepared two lipid-solid dispersion of NCTD-loaded alginate/CS microspheres (LSD/NTCD-ACMs), which showed a well-sustained release profile after a mild burst release. LSD/NTCD-ACMs administration via the hepatic artery can also achieve better therapeutic effect than that of NCTD solution in the VX2 liver cancer model, with higher degree of hepatocyte necrosis, longer survival time and less toxic side effects. Therefore, LSD/NTCD-ACMs are potential candidates for embolization of liver cancer.

Silk fibroin is a biomedical material extracted from silk with good biodegradability and biocompatibility, as well as non-toxic, non-sensitizing and non-irritating effects to the body. It can be used as a carrier material for sustained drug release to improve the bioavailability of drugs [170, 171]. Zhang et al. [172] prepared a NCTD-loaded silk fibroin/CS microsphere (NCTD-SF/CS-MS) using silk fibroin and CS as carriers by the emulsification-gelation method. The microsphere had an average diameter of (184 ± 5) µm. The release profile of NCTD-SF/CS-MS followed Weibull distribution in vitro and sustained for about 14 days, better than NCTD-CS-MS. Therefore, the tumor necrosis area and the life prolonging rate in NCTD-SF/CS-MS group was better than that in NCTD-CS-MS group, and angiograms showed a complete occlusion with litter collateral formation. Wen et al. [173, 174] also prepared a NCTD-N-CS/silk fibroin microsphere (NCTD-N-CS/SF-MS) through the emulsification-gelation method. NCTD-N-CS/SF-MS had an average size of (117 ± 4.3) µm. The releasing test in vitro manifested that 60% of NCTD was steadily released in 7 days. The tumor inhibition rate and tumor cell necrosis rates in NCTD-N-CS/SF-MS group was 85.01% and 56.78%, respectively, better than that in blank N-CS/SF-MS + NCTD solution groups. 30 days after TAC, CT imaging showed that the NCTD-N-CS/SF-MS group had smaller tumor volume, more pronounced necrosis area and longer survival time (36.25 days) than other groups. Angiograms showed a complete occlusion with collateral formation.

## New developments of NCTD drug delivery system

### Dual drug-loaded drug delivery system-based NCTD delivery

Compared with single drug therapy, the strategy of multi-drug combination therapy can simultaneously act on multiple pathways and multiple targets to exert synergistic antitumor effects, reduce the toxicity and side effects caused by a single drug, and overcome treatment-related multidrug resistance (MDR), and so on. However, the different physicochemical properties and pharmacokinetic properties of drugs may lead to the inability of drugs to reach tumor cells synchronously to exert antitumor effects [175]. The nanocarrier-mediated multidrug delivery system can improve the deficiencies of existing antitumor multidrug delivery strategies, and deliver the drugs to tumor cells synchronously in the best synergistic ratio, thereby enhancing drug efficacy and reducing toxicity [176, 177]. Several studies have shown that the co-loading of NCTD and other chemotherapeutic drugs such as oleanolic, tetrandrine (Tet) and ABT-737 in the same nanocarrier can significantly improve the antitumor effect of chemotherapeutic drugs, reduce the toxic and side effects of chemotherapeutic drugs, and reverse MDR.

Oleanolic acid is a pentacyclic triterpenoid compound, which has a certain inhibitory effect on various cancers such as liver cancer, colon cancer, and breast cancer [178]. Liu et al. [179] developed dual-drug liposomes containing NCTD and oleanolic using film hydration method. The EE of NCTD and oleanolic were 45.6% and 84.5%, respectively.

**Fig. 9:**
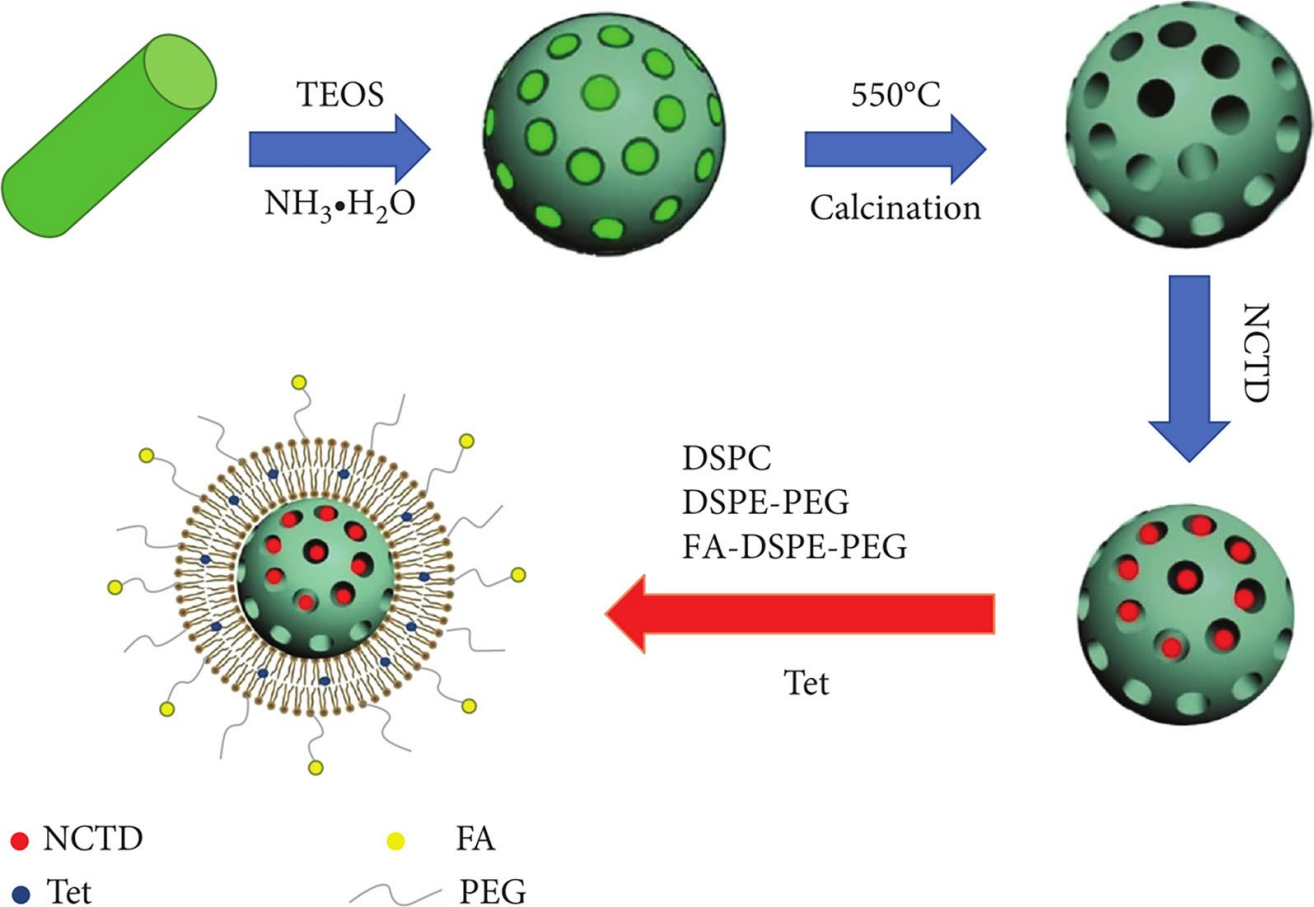
The synthetic route of FA-LP@Tet/(MSNs@NCTD). Reproduced with permission from reference [182]. Copyright 2019, HINDAWI

Tet, one of the main active ingredients of *Stephania tetrandra* S. Moore, is a bisbenzylisoquinoline alkaloid with broad-spectrum antitumor activity [180]. Xiong et al. [181] prepared dual-drug liposomes using NCTD-mesoporous silica nanoparticles (MSN-NCTD) and Tet. The dual drug loaded liposomes had uniform particle size of (207.5 ± 3.6) nm, zeta potential of (1.345 ± 0.173) mV and high EE (86.62% and 79.19% respectively for NCTD and Tet), showing sustained drug release characteristics. In addition to its own antitumor activity, TET is also a reversal agent for MDR of tumors [180]. Xiong et al. [182] constructed a FA receptor-targeted NCTD/Tet dual-drug loaded lipid nanoparticles [(FA-LP@Tet/(MSNs@NCTD)] based on MSNs, with an average size of (153.17 ± 3.17) nm (Fig. 9). The FA modification significantly increased intracellular uptake of FA-LP@Tet/(MSNs@NCTD) on HepG2 cells. Moreover, FA-LP@Tet/(MSNs@NCTD) could reverse MDR by inhibiting P-gp in HepG2/Adr cells.

**Fig. 10:**
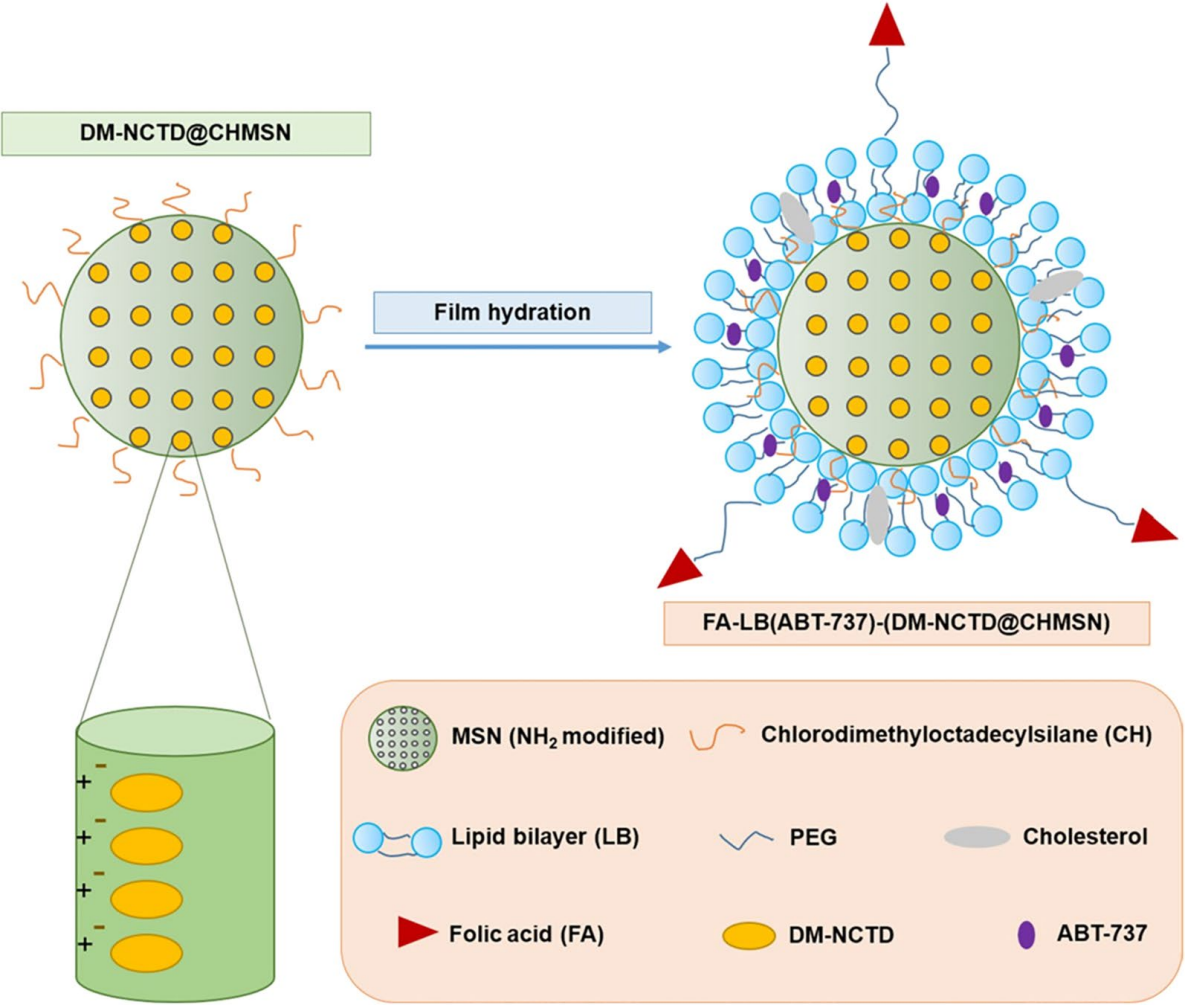
Schematic illustration of the synergistic co-delivery of diacid metabolite of norcantharidin and ABT-737 based on folate-modified lipid bilayer-coated mesoporous silica nanoparticle. Reproduced with permission from reference [184]. Copyright 2020, BIOMED CENTRAL

ABT-737 is an antagonist of small molecule Bcl-2, which could induce tumor cell apoptosis without causing damage to normal cells. NCTD combined with ABT-737 has a synergistic effect on the treatment of HCC [183]. Liu et al. [184] prepared a FA-lipid bilayer (LB)-chlorodimethyloctadecylsilane (CH)-coated MSN (FA-LB-CHMSN) with diacid metabolite of NCTD (DM-NCTD) loaded in CHMSN and ABT-737 loaded in lipid bilayer [FA-LB(ABT-737)-(DM-NCTD@CHMSN)] (Fig. 10). This nanoparticle enhanced intracellular uptake of the drugs through FA receptor-mediated endocytosis, thereby inducing marked cell apoptosis on H22 cells and showing significant antitumor activity on H22 tumor model, with no apparent systemic toxicity.

### Exosomes-based NCTD delivery

**Fig. 11:**
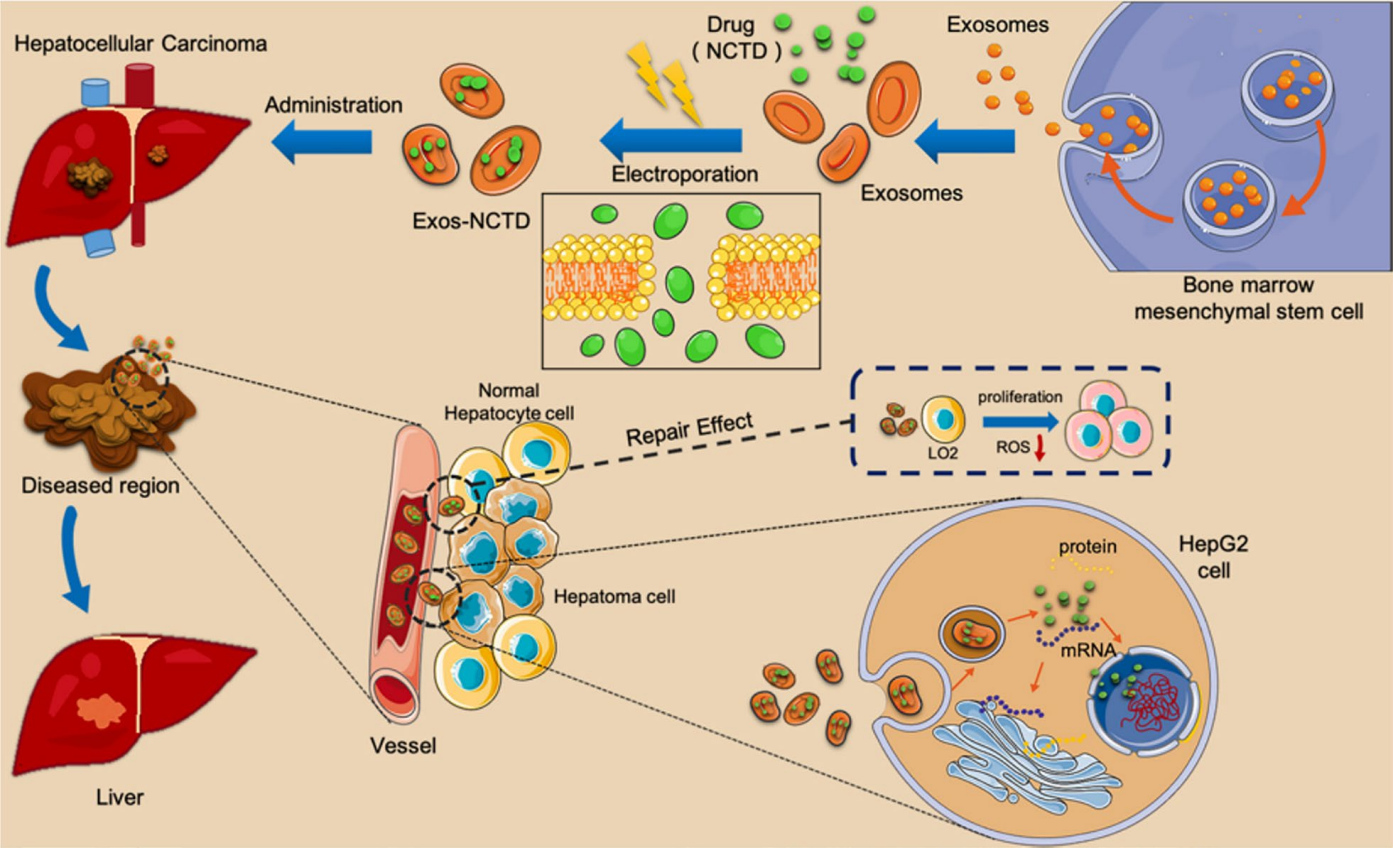
Schematic illustration of norcantharidin encapsulated exosomes derived from bone marrow mesenchymal stem cells for the treatment of hepatocellular carcinoma. Reproduced with permission from reference [187]. Copyright 2021, American Chemical Society

Exosomes are one of the most important ways of cell-to-cell communication in living lives. Due to its excellent compatibility, good permeability, natural stability, and low immunogenicity and toxicity, exosomes have attracted the attention of many researchers in recent years as a new drug delivery system [185]. Yang et al. [186] prepared an NCTD-loaded rat serum exosome. The optimal process conditions for exosome-embedded NCTD were: NCTD level was 0.4 mg/mL, incubation temperature was 30.81 ℃, and incubation time was 3.28 h. The average EE of NCTD was 15.36%, and the average particle size was 97.45 nm. Mesenchymal stem cell-derived exosomes (MSC-Exos), nanoscale lipid bilayer multivesicular bodies (40–100 nm) that are secreted by MSCs under physiological or pathological conditions, also have potential as NCTD delivery vehicles for HCC therapy. Liang et al. [187] prepared an NCTD-loaded bone mesenchymal stem cell-derived exosomes (BMSC-Exos) (BMSC-Exos-NCTD) via electroporation, with an average particle size of 127 nm and in vitro sustained drug release properties. BMSC-Exos-NCTD significantly enhanced cellular uptake, induced cell cycle arrest, inhibited tumor cell proliferation, increased apoptosis, and exerted excellent in vivo antitumor activity with no apparent systemic toxicity compared with the free NCTD. Furthermore, the BMSC-Exos carrier has an in situ homing effect on the tumor sites of HCC in mice. BMSC-Exos-NCTD also repaired damaged liver tissues in liver sections, as reflected by the increase in cellular proliferation and the inhibition of liver cell oxidation on the normal liver cell line L02. Therefore, BMSC-Exos, as drug delivery systems, have great potential in the HCC treatment in combination with NCTD (Fig. 11).

## Conclusion

NCTD, a synthetic derivative of CTD, is a promising anticancer drug first developed in China for the treatment of liver cancer. Compared with CTD, it not only significantly improves the anticancer effect, but also greatly reduces the nephrotoxicity and the strong irritation to the urinary system. NCTD used clinically is mainly in tablets and injections. It has unique advantages in the treatment of tumors, including increasing white blood cells, regulating immunity without producing bone marrow suppression, and so on. In many cases, it is the first-choice adjuvant drug for various cancers such as liver cancer, esophagus cancer, gastric cancer and cardia cancer, especially for primary liver cancer, and it can be used before and after surgery, radiotherapy and chemotherapy. NCTD can also improve liver function and can be used to treat hepatitis, cirrhosis and hepatitis B virus. These advantages make NCTD have a good application prospect. Mechanically, NCTD can inhibit proliferation through inhibiting the Wnt/β-catenin, epidermal growth factor receptor (EGFR) and c-Met pathways, and suppressing the activity of Protein phosphatase 5 (PP5) phosphatase [188–190]. NCTD can also inhibit proliferation through cell cycle arrest and inhibition of DNA replication by blocking protein kinase B (Akt) and extracellular signal-regulated kinase (ERK) signaling, inducing cell division cycle 6 (Cdc6) degradation and regulating the expression of cyclins, cyclin-dependent kinases (CDKs) and cyclin-dependent kinase inhibitors [191–193]. Moreover, NCTD can induce tumor cell apoptosis by promoting ROS production, regulating the caspase-mitochondrial pathway and mitogen-activated protein kinase (MAPK)-related pathways, blocking the phosphatidylinositol 3-kinase (PI3K)/Akt/nuclear factor-kappaB (NF-κB) pathway, decreasing the Bcl-2 and survivin expression, and increasing the caspases, p53 and Bax expression [194–198]. NCTD can also induce apoptosis and inhibit proliferation via suppressing the activity of PP2A [199, 200]. NCTD can inhibit the invasion and metastasis through reducing the activity of MMP-2/-9 by upregulating the signal transducer and activator of transcription 1 (STAT1) and inhibiting the transactivation of Sp1, and inhibit the epithelial-mesenchymal transition (EMT) process via blocking the αvβ6-ERK-Ets1 and YAP pathways [201–203]. NCTD can inhibit angiogenesis by downregulating the expression of VEGF, VEGFR-2, angiopoietin-2 (Ang-2), and upregulating the expression of thrombospondin (TSP) and TIMP-2 [204, 205]. NCTD can inhibit the tumor vasculogenic mimicry via suppressing MMP-2 expression and blocking the Ephrin Type a Receptor 2/Focal Adhesion Kinase/Paxillin pathway [206, 207]. NCTD can also enhance the anti-vasculogenic mimicry activity of TIMP-2 by downregulating MMP-2 and membrane type 1-MMP (MT1-MMP) expression [208]. NCTD can inhibit lymphangiogenesis by downregulating the expression of VEGF-A/-C/-D and VEGFR-2/-3 [209]. NCTD can overcome MDR by inhibiting the MDR-1/P-gp and Mcl-1 expression, the Met/PI3K/Akt pathway and the sonic hedgehog (Shh) signaling [210–212]. Furthermore, NCTD can positively regulate macrophage-mediated immune responses via the Akt/NF-κB pathway, and can decrease the number of tumor-infiltrating Tregs and increase the number of CD4 + and CD8 + T cells [213, 214].

However, although NCTD greatly reduces the toxicity of CTD, there is still a certain degree of urinary toxicity and organ toxicity. For example, Li et al. [215] found that intraperitoneally administered with NCTD (0.8 mg/kg, 1.6 mg/kg and 6.0 mg/kg) every 24 h for 12 weeks could increase ALT, AST, albumin (ALB), ALP liver function indexes and creatinine (CRE), blood urea nitrogen (BUN) kidney function indexes. Microscopic examination showed that liver, lung and kidney had different degrees of pathological changes, and the toxicity changes showed a certain time and dose correlation. Fan et al. [216] showed that after NCTD (10 mg/kg) was administered by gavgae for 2 weeks, the contents of ALT and BUN in serum of mice were abnormally increased; and NCTD could lead to inflammatory cell infiltration in liver, vacuolar changes in liver cells, and diffuse damage of glomeruli and tubules. Martínez-Razo G et al. [217] showed that intraperitoneally administered with NCTD (3.0 mg/kg and 6.0 mg/kg) every 24 h for 6 days significantly modified the phosphorylase, alanine transaminase, and γ-glutamyl transferase activities. Histopathological analysis revealed a significant elevation in hepatocytes’ nuclei average size and total area (3 mg/kg), as well as centrilobular vein and adjacent sinusoidal capillaries showed a significant difference. The portal triad presented a significant difference in veins and capillarity count in 6 mg/kg. Renal samples showed cortex convoluted tubules’ average size significantly augmented in both doses’ groups, and tubule count was found augmented in 6 mg/kg. The mechanism of NCTD toxicity needs to be further studied. In addition, the poor solubility, short half-life, fast metabolism, as well as high venous irritation and weak tumor targeting ability limit its wide clinical application. Researchers at home and abroad have tried to solve these problems by means of preparations, and have made great progress in recent years. NCTD-loaded passive targeted drug delivery systems including liposomes, micelles, nanoparticles, microemulsion and self-microemulsion, polymer-conjugated drug delivery systems and microspheres, significantly improved the solubility and in vivo pharmacokinetics of NCTD, and could passively accumulate NCTD into tumor tissues by EPR effect, and could release the drug slowly and continuously, thereby greatly enhancing the antitumor effect and reducing the toxicity of NCTD. Moreover, monoclonal antibodies or ligands-modified drug delivery systems could deliver NCTD to tumor cells more precisely through highly expressed antigens or receptors on the cell surface, reducing the distribution of drugs in normal tissues, thereby further enhancing the antitumor effect and reducing the toxicity of NCTD. NCTD-loaded drug delivery systems designed and constructed based on physicochemical characteristics such as pH, temperature, charge and tumor blood vessels are of great significance in controlling drug release at target sites. The multi-drug co-loading nano-delivery system developed by NCTD combined with other chemotherapeutic drugs such as oleanolic acid, tetrandrine and ABT-737 can simultaneously deliver multiple drugs to tumor cells, which can not only enhance the antitumor effect of chemotherapeutic drugs, but also reduce the toxicity and side effects of chemotherapeutic drugs, and has the effect of reversing the multidrug resistance of tumors, showing a good application prospect. Besides, as a new drug delivery system, exosomes endow NCTD with better biocompatibility and immune evasion ability, providing new ideas for personalized treatment of tumor patients; in the future, other biomimetic nano-delivery systems such as erythrocyte membrane, tumor cell membrane, and endothelial cell membrane-coated nanoparticles can be further developed, thereby opening up new directions for NCTD delivery.

Among the numerous targeted drug delivery systems, nanohydrogels that can be used for topical drug delivery are a very promising drug delivery system. Clinically, surgical resection is still the main means of tumor treatment, but the tumor resection process is often accompanied by residual tumor cells. According to statistics, about 90% of cancer patients eventually die because of tumor recurrence or metastasis [218, 219]. Immediate implantation of controlled release nanohydrogels into surgical wounds is an effective strategy to prevent tumor recurrence. Chemotherapy and radiotherapy are often used after surgery to prevent tumour recurrence and metastasis, but these therapies often cause toxicities [220, 221]. The nanohydrogel can efficiently load and deliver drugs, and make the drugs concentrated near the residual tumor tissue for a long time and slow release, which greatly reduces the toxic and side effects of drugs. Moreover, the hydrogel has good plasticity and is suitable for surgical wounds of any shape. It can also absorb local bleeding during tumor removal, prevent the spread of tumor cells and wound infection [222–225]. In addition, the modified nanohydrogel can also achieve on-demand drug release through light response, magnetic response, ultrasonic response, electrical response, pH response, ROS response, enzyme response and MMPs response [226–229]. Furthermore, some nanomaterials can give hydrogels luminescence and imaging functions to better monitor drug release behavior [230, 231]. For example, Zhu et al. [232] developed an injectable MMPs- and ROS-responsive hydrogel. These hydrogels exhibited postoperative environmental responsiveness and achieved sustained temozolomide (TMZ) release in the surgical cavity. The anti-glioma effects in the incomplete operation models of C6 and U87 glioma indicated that these hydrogels effectively inhibited postsurgical glioma recurrence while minimizing systemic toxicity. Wu et al. [233] developed a tumor-targeted nanocomposite double-network hydrogel by NIR-induced polymerization of polyethylene glycol acrylate (PEGDA) and endogenous Ca^2+^-crosslinked alginate with the addition of radioisotope-labeled ^125^I-GNR-RGDY. The hydrogel exhibited excellent photothermal therapeutic efficacy and brachytherapy after in situ injected into the cavity of postoperative breast cancer tissue. Furthermore, photothermal ablation could simultaneously eliminate potential pathogenic bacteria to prevent postoperative wound infection. Notably, the embedded ^125^I-GNR-RGDY also endowed the hydrogel with long-term isotope-imaging properties. Yan et al. [225] prepared an in situ formed magnetic hydrogel with promising bioapplicable thermal-responsiveness, strong adhesion in wet conditions, high magnetic hyperthermia, and efficient hemostasis function, which effectively reduced the recurrence rate of liver cancer after surgery. Chen et al. [234] also developed an in situ formed immunotherapeutic bioresponsive gel. The fibrinogen solution containing anti-CD47 antibody-loaded CaCO_3_ nanoparticles and thrombin solution can be quickly sprayed and mixed within the tumour resection cavity after surgery to form the gel in situ. CaCO_3_ nanoparticles can gradually dissolve and release the encapsulated aCD47 in the acidic and inflamed tumor microenvironment, thus promoting the activation of M1-type tumor-associated macrophages (TAMs), inducing macrophage phagocytosis of cancer cells via blockade of the CD47 and signal regulatory protein-α (SIRPα) interaction as well as boosting antitumor T cell responses. After treatment, 50% of the melanoma mice survived at least 60 days without tumor recurrence, while none of the control mice survived longer than 30 days. NCTD can inhibit and kill tumor cells through multiple pathways and multiple targets, which is a promising antitumor drug. Therefore, the research and development of NCTD nanohydrogels is of great significance for inhibiting tumor recurrence after surgery.

Although there are many studies on NCTD-targeted drug delivery systems, there are still many problems to be solved before these drug delivery systems are applied to clinical practice. For example, the research on NCTD loaded liposomes should pay attention to the low EE of drugs, leakage during storage, sterilization stability and other problems arising in the process of industrialization. Microemulsion can well solve the problems of high irritation and fast elimination of NCTD, but screening of safe and efficient surfactants should be a focus of research. NCTD microspheres can enhance the anti-HCC effect by embolization and sustained release of microspheres, and the embolization microspheres have large particle size and high drug loading, which has a very broad prospect for the treatment of HCC. However, the preparation process of microspheres still need further research to meet the requirements of high drug loading and high EE. In addition, the drug release behavior, solvent residual toxicity and drug stability of microspheres also need to be further studied. With the continuous emergence of new nanomaterials and the in-depth research on the preparation, physical and chemical properties and biological properties of nanoparticles, the nanoparticle drug delivery system has made great progress in improving the tumor targeting efficiency of NCTD, but the sterilization and storage stability, degradation characteristics, drug release characteristics, drug loading, EE and surface modification of nanoparticles, and the solvent residual toxicity of nanoparticles and the safety of nanomaterials in vivo should be further explored [235]. If the above problems can be continuously solved in the research, it will be very conducive to the development and clinical application of NCTD targeted drug delivery system.

## Data Availability

All data generated or analyzed during this study are included in this published article and the Additional Information.
